# Multilayer Adjuvanted Influenza Protein Nanoparticles Improve Intranasal Delivery and Antigen-Specific Immunity

**DOI:** 10.1021/acsnano.4c14735

**Published:** 2025-02-15

**Authors:** Jaeyoung Park, Thomas Pho, Noopur Bhatnagar, Linh D. Mai, Mariela R. Rodriguez-Otero, Surya Sekhar Pal, Chau Thuy Tien Le, Sarah E. Jenison, Chenyu Li, Grace A. May, Marisa Arioka, Sang-Moo Kang, Julie A. Champion

**Affiliations:** a School of Chemical and Biomolecular Engineering, Georgia Institute of Technology, Atlanta, Georgia 30332, United States; b Bioengineering Program, Georgia Institute of Technology, Atlanta, Georgia 30332, United States; c Center for Inflammation, Immunity & Infection, Institute for Biomedical Sciences, Georgia State University, Atlanta, Georgia 30302, United States; d Department of Chemistry, Tokyo University of Science, Shinjuku-ku, Tokyo 162-8601, Japan

**Keywords:** influenza vaccine, intranasal, nanoparticle, biodistribution, layer-by-layer, subunit vaccine, mucosal immunology

## Abstract

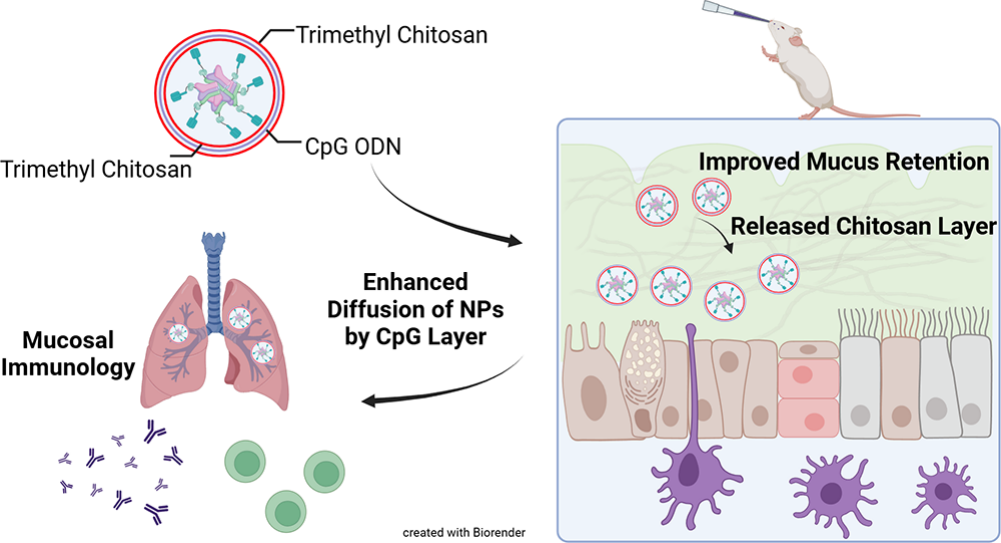

Intranasal vaccination is a desired route for protection against influenza viruses by mucosal and systemic immunity. However, the nasal mucosa impedes the intranasal delivery of vaccines. Here, we formulated layer-by-layer (LBL) influenza vaccine nanoparticles for effective intranasal delivery by coating them with alternating mucoadhesive cationic chitosan and muco-inert anionic CpG adjuvants. The nanoparticle cores were formed by desolvating influenza M2e antigen and coating it with hemagglutinin (HA) antigen via biotin–streptavidin conjugation. LBL modification promoted nasal delivery and interaction with the resident immune cells. Intranasal administration with LBL nanoparticles significantly improved cellular and humoral immune responses against HA and M2e including high IgA titers, a hallmark of potent mucosal immunity and persistence of immune responses. Distinct trends for antigen-specific immune responses were observed for different routes of vaccination. The enhanced immune responses conferred mice protection against the influenza challenge and prominently reduced viral titers, demonstrating the effectiveness of intranasal LBL vaccine nanoparticles.

## Introduction

Vaccination has been an effective strategy to curb the spread of respiratory diseases.^1^ While the prophylactic effectiveness may be substantially affected by the route of delivery, intramuscular (IM) injections are the most common to stimulate systemic immune responses against influenza.^2^ However, localized immunity in the respiratory tract where influenza infection is initiated is a rational and promising strategy to promote the neutralization of pathogens in mucosal tissues before systemic circulation.^3−5^ Previous studies have shown that secretory IgA antibodies induced by intranasal (IN) administration of influenza vaccines provided effective virus neutralization and cross-reactive immune responses against influenza.^6−8^ It was also reported that human subjects immunized IN with live attenuated influenza vaccine (FluMist) showed 85% protective efficacy, while IM injection of inactivated influenza vaccine resulted in 71% protective efficacy.^9,10^ However, FluMist provided less effective prophylactic activity against influenza A(H1N1)pdm09 (A/California/2009) than IM injection of inactivated influenza vaccine, and therefore, the IN vaccine was not recommended from 2016 to 2018.^11^ The inconsistent effectiveness of the IN influenza vaccine could have been affected by vaccine components and, thus, be improved by enhancing the immunogenicity of IN vaccines. Some studies have demonstrated that highly immunogenic antigens or adjuvants are necessary for IN vaccines to be effective.^7,12−14^ Additionally, IN vaccine immune responses are often short-lived.^15,16^ This limitation arises from factors such as the clearance of vaccines by physiological barriers and the rapid turnover time of mucus, which reduce the likelihood of antigen uptake by immune cells in the respiratory mucosa. Since both efficacy and durability are desired, attention should be paid to vaccine design and formulation such as appropriate vaccine adjuvants, increased retention time of vaccine particles at the site of immune activity, high diffusion of vaccine particles in mucus for antigen delivery to mucosal immune cells, and design of particles for enhanced antigen presentation to immune cells.

One approach to formulate IN vaccines is to use cationic nanoparticles (NPs) for enhanced electrostatic attraction toward negatively charged components of mucus, such as oligomeric glycoproteins, increasing mucus adhesion.^17,18^ The prolonged retention of such mucoadhesive NPs in mucus allows them to effectively activate mucosal immunity. Chitosan is an attractive candidate for synthesizing cationic NPs as it is not only a mucoadhesive polymer^18^ but also an adjuvant.^19^ However, the nasal mucosa also presents physical–biological barriers to prevent pathogen access, and these also impede IN vaccine delivery. The mucoadhesive NPs may not be able to penetrate through the mucus layer and epithelium before being eliminated by mucus turnover. Therefore, materials to activate the immune system in the respiratory mucosa must first diffuse through mucus layers and then engage cellular uptake and transport across epithelial cells before the 15–30 min mucociliary clearance mechanism.^20,21^ A strategy to improve mucosal delivery of vaccines includes coating of nanocarriers with muco-inert hydrophilic polymers such as polyethylene glycol (PEG).^22^ The formulation of NPs with short dense neutral PEG (<2 kDa) coatings on the surface improved diffusion across the negatively charged mucus by reducing hydrophobic and electrostatic interactions. However, PEG-mediated coating of NPs may shield antigen epitopes from presentation and hinder internalization of NPs, as found with PEG coated human adenovirus serotype 5 when delivered either IM or IN.^23^ Furthermore, PEG can induce allergic reactions or even life-threatening anaphylaxis.^24−27^ Anti-PEG antibody immune responses were previously reported in humans after SARS-CoV-2 mRNA vaccine was given, necessitating further research to study the impact of anti-PEG immune responses on reactogenicity.^28^ Although PEGylated mRNA lipid NPs were also shown to induce severe anti-PEG antibody immune responses in mice, the antibody production against PEG can be largely affected by routes of administration and the rate of shedding PEG from NPs, with the intramuscular route or fast shedding eliciting generally low levels of anti-PEG antibody titers. Therefore, further efforts need to be made to optimize the formulation of PEGylated NPs and vaccination strategy.^29,30^ An anionic cytosine phosphorothioate guanine oligodeoxynucleotide (CpG ODN) could be an alternative to PEG as coating NPs with hydrophilic DNA has shown to improve the diffusion of NPs in mucus.^31^ CpG ODN is an FDA-approved adjuvant for human vaccine use^32−34^ that has been extensively investigated in IN vaccine formulations due to its reported effectiveness as an IN adjuvant.^12,13,35^

To improve mucoadhesive and mucopenetrating properties of NPs, both muco-inert and mucoadhesive polymers can be exploited. We reported that a layer-by-layer (LBL) approach, which coats NPs with alternating charged layers of chitosan–CpG–chitosan, improved porcine mucus diffusion. The outer mucoadhesive chitosan layer was released during mucus interaction and consecutively exposed a muco-inert layer of anionic oligonucleotides, rendering NPs diffusive through mucus.^36^ In addition to the enhanced NP delivery in mucus, LBL can be an effective strategy to improve immune responses. A number of studies utilized LBL with charged adjuvants or immunomodulators and peptide antigens to form NPs for promoting immune activation or tolerance, known as polyelectrolyte multilayers (PEMs).^37−40^ When mice were immunized with PEMs loaded with anionic polyIC (Toll-like receptor 3 agonist) and ovalbumin CD8^+^ T-cell peptide (SIINFEKL) with appended cationic arginine anchor, CD8^+^ T-cell responses were significantly enhanced compared to a soluble mixture of polyIC and SIINFEKL.^37,40^ In this work, we compare LBL-coated and PEG-coated ovalbumin (OVA) NPs for their nasal biodistribution and humoral immune responses. Both were improved over uncoated NPs, although PEGylated OVA NPs induced anti-PEG antibodies and were not used further for influenza antigens.

When designing IN vaccines, the choice of the antigen format or vaccine platform is also critical. In 2024, the FDA approved FluMist for at-home use to promote accessibility for influenza immunization, leveraging its noninvasive and user-friendly IN administration method.^41^ However, prior exposure to influenza viruses or antigens can negatively affect vaccine efficacy, particularly for whole-pathogen platforms like FluMist.^42−46^ This phenomenon, known as antigenic imprinting or original antigenic sin, makes FluMist less effective in adults compared to children. To address these challenges, subunit vaccines, which use viral components to generate vaccine nanoparticles, present a promising alternative for IN vaccine development. In this study, we selected a subunit vaccine platform to evaluate the function of the LBL coating on influenza vaccine NPs for effective IN delivery and vaccination. To fabricate influenza vaccine NPs, the tetrameric chimeric influenza A virus matrix protein 2 ectodomain (M2e) was desolvated to form nanoclusters, which were then surface functionalized with multiple copies of trimeric hemagglutinin (HA) via site-specific biotin–streptavidin conjugation (HA-4M2e NPs). This design maintained the functional structure of HA and induced significantly improved mucosal and systemic immune responses when LBL was coated with chitosan and CpG and administered IN. Different routes of vaccination were explored and displayed distinct trends of immune responses against HA and M2e. This work demonstrates the importance of designing IN vaccines for both delivery and immunogenicity and provides a general approach that could be applied to many kinds of vaccine NPs for which mucosal immunity is desired.

## Results

### Layer-by-Layer Nanoparticles Exhibit Enhanced Nasal Delivery

Prior to fabricating and immunizing with influenza vaccine NPs, we used model ovalbumin (OVA) vaccine NPs to evaluate the impact of the LBL coating on IN delivery. These particles demonstrated a unique bimodal diffusion distribution profile with highly diffusing and slowly diffusing populations resulting in an average greater diffusion in porcine mucus than PEGylated OVA NPs, implying that the dynamic shedding of noncovalent layers promotes diffusion.^36^ OVA NPs were used as a control since soluble OVA antigens were shown to diffuse rapidly out of the nasal cavity before 1 h upon intranasal administration.^47^ OVA protein was conjugated with Alexa Fluor 647 NHS Ester for a degree of labeling of ∼1.0. OVA NPs including 15% fluorescently labeled OVA were synthesized using desolvation with ethanol and stabilized using DTSSP (3,3′-dithiobis(sulfosuccinimidyl propionate)) cross-linking (Figure S1). LBL OVA NPs were synthesized by coating the OVA NPs with two layers of short trimethyl chitosan and a layer of CpG ODN 1826 between chitosan layers as the cationic and anionic polyelectrolytes. Alternating surface charge at each step confirmed successful coating (Figure S1b). As demonstrated in Figure S1c, LBL OVA NPs activated signaling cascades for Toll-like receptor 9 (TLR9) in HEK293 reporter cells, indicating that the CpG layer remains intact and functional as an adjuvant in LBL OVA NPs. For the HEK-Blue hTLR9 cell assay, 100 μg/mL CpG was used as a positive control as per the manufacturer’s instructions. An increase in TLR9 activation signals was observed as the concentration of LBL OVA NPs rose from 0.125 μg/mL (containing 0.034 μg/mL CpG) to 1 μg/mL (containing 0.269 μg/mL CpG), demonstrating a dose-dependent activation of TLR9. Notably, 1 μg/mL LBL OVA NPs (0.269 μg/mL CpG) induced TLR9 activation comparable to that triggered by 100 μg/mL CpG alone, indicating that a lower amount of CpG in the LBL OVA NP format is sufficient for robust TLR9 activation. PEGylated OVA NPs (PEG OVA NPs) were synthesized via NHS-ester conjugation of a short ∼750 Da PEG onto the surface to promote antifouling properties in mucus.^36,48−50^ PEGylation of the external surface of OVA NPs was confirmed by measuring the surface charge to be neutral (−6.89 ± 0.4 mV) as shown in Figure S1b. Neither surface modification altered the diameter or polydispersity index of the OVA NPs (Figure S1a).

**Figure 1:**
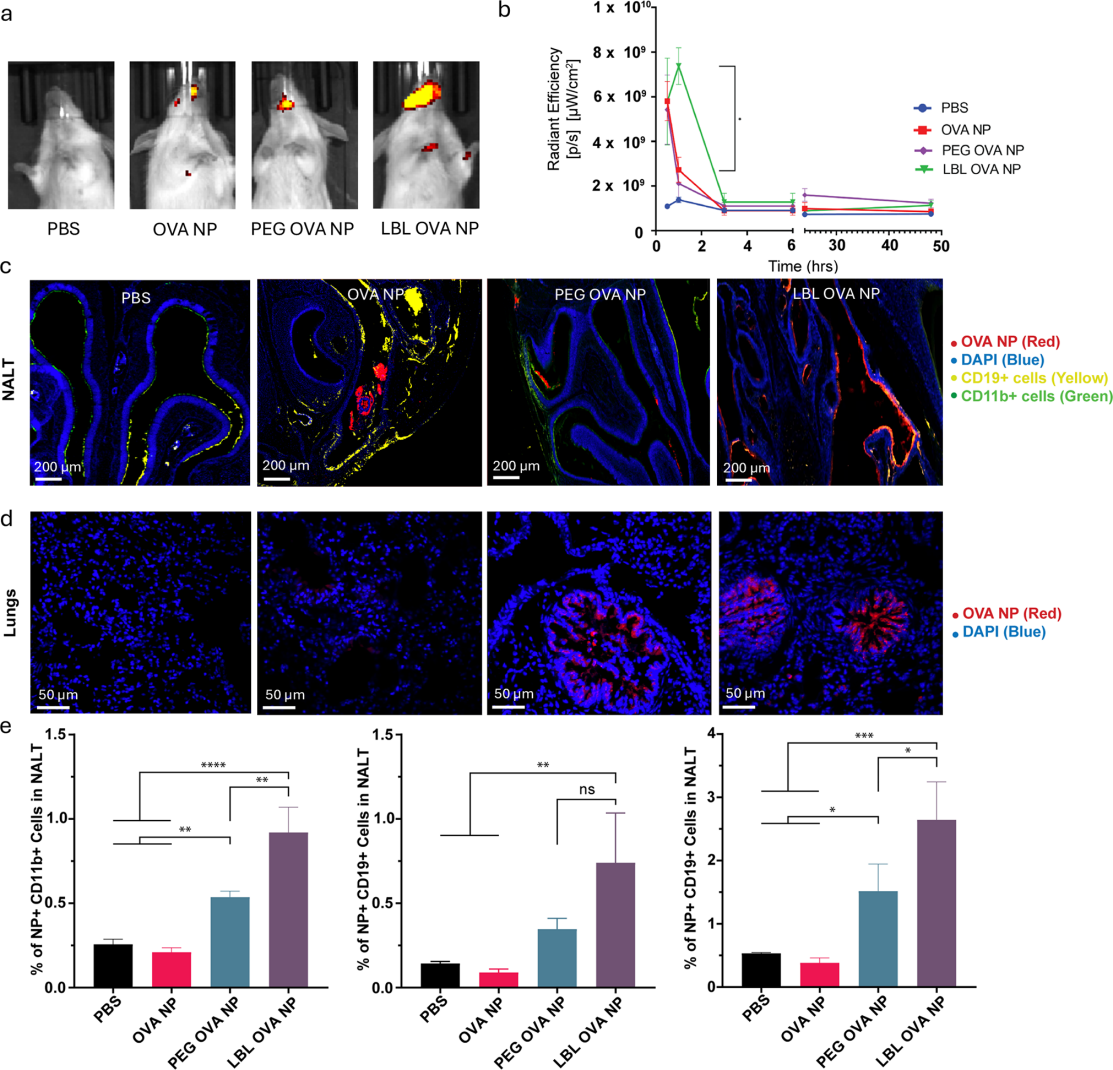
*In vivo* biodistribution study. (a) IVIS spectrum images of mice administered IN with PBS, OVA NPs, PEG OVA NPs, and LBL OVA NPs 1 h after administration. (b) Profiles of radiant efficiency in mice over time. (c, d) Histology of NALT 24 h after nasal administration and lung stained with DAPI (blue) for cells, PE anti-CD19 for B cells (yellow), and FITC anti-CD11b for leukocytes (green) and biodistribution of PBS, OVA NPs (red), PEG OVA NPs (red), and LBL OVA NPs (red). (e) Uptake of OVA, PEG OVA, and LBL OVA NPs isolated from NALT by single cells, which were stained by anti-CD11b (biomarker for DCs) antibody or anti-CD19 (biomarker for B cells) antibody, and percent population of CD19^+^ cells isolated from NALT of mice treated with NPs. The brightness and contrast of histology images were uniformly adjusted to improve visualization of the red fluorescence of the NPs. The *p* values (*n* = 3) were determined by one-way ANOVA with Tukey’s *post hoc* multiple comparison analysis: ns for not significant, * for ≤0.05, ** for ≤0.01, *** for ≤0.001, and **** for ≤0.0001.

Fluorescently labeled OVA NPs (5 μg) were administered IN to mice, and retention in the nasal cavity was evaluated using the IVIS Spectrum in vivo imaging system. We observed high fluorescence levels of LBL OVA NPs in the nasal cavity after 1 h compared to both PEG and unmodified OVA NPs as shown in Figure 1a,b. At 3 h, none of the NPs exhibited detectable signals by IVIS. To improve the sensitivity and resolution of NPs in the nasal cavity, we utilized multispectral imaging on whole head (medial cut) and lung sections collected 24 h after administration and labeled by immunohistochemistry (CD11b for dendritic cells (DCs), CD19 for B cells, DAPI for all nuclei). Optimization of histological processing and use of tyramine signal amplification enabled visualization of NPs in tissue down to the single cell level. The images revealed that all NPs were present in nasal tissue at 24 h, long after the IVIS signal was undetectable. However, the distribution of NPs was different (Figure 1c). Unmodified NPs appeared highly aggregated and primarily in lumens, not colocalized with cells. Conversely, the LBL NPs, and to a slightly lesser extent the PEG NPs, appeared distributed along the lumen cell boundary in regions enriched with B cells and DCs. LBL and PEG OVA NPs both reached the lungs and similarly concentrated at the cell layer of lumens (Figure 1d). Consistent with *ex vivo* mucus diffusion experiments where LBL enhanced NP diffusivity,^36^ LBL coating enhanced NP delivery to both the nasal cavity and lungs. To determine if NPs penetrated the mucus and reached NALT, 6 h after delivery, NALT was removed and mechanically homogenized, and single cells were isolated, stained for CD11b and CD19, and evaluated by flow cytometry for uptake of fluorescently labeled NPs (Figure 1e). The results demonstrated that LBL OVA NPs had the highest uptake in DC populations, while LBL and PEG OVA NPs had similar B-cell uptakes, indicating that LBL coating was a promising formulation for improved uptake of NPs by APCs in the nasal cavity. The highest uptake of LBL NPs by CD11b^+^ myeloid DCs implies that potent antigen-specific adaptive immunity can be elicited by LBL NPs as CD11b^+^ DCs play a critical role in inducing the production of Th1 and Th2 cytokines^51,52^ and can migrate to the nasal passage for eliciting antigen-specific immune responses.^53,54^ Furthermore, PEG and LBL OVA NPs recruited more B cells (CD19^+^) in the nasal cavity compared to those of the OVA NPs (Figure 1e). Based on the results, potent humoral immune responses can be expected from PEG and LBL OVA NPs. Several studies have highlighted the importance of surface modified NPs for optimizing the mucus diffusivity of NPs, which significantly affects their mucus penetration and retention.^22,55−57^ However, PEGylated vaccines can induce an anti-PEG humoral immune response, which can facilitate clearance and promote hypersensitivity reactions.^58,59^ Layering NPs with polyelectrolytes offers unique mucus diffusion properties to behavior as both a mucoadhesive and mucus-penetrating carrier. The ability of LBL NPs to overcome the mucus barrier and reach local B cells and DCs in the NALT motivates the characterization of the immunogenicity of LBL NPs in comparison to PEG NPs.

BALB/C mice were vaccinated and boosted 1 month later IN with LBL, PEG, and unmodified OVA NPs plus a soluble formulation containing OVA and the LBL adjuvants chitosan and CpG. Consistent with the higher percent populations of CD19^+^ cells in mice treated with PEG and LBL OVA NPs than with OVA NPs (Figure 1e), mice vaccinated with LBL OVA NPs and PEG OVA NPs showed enhanced IgG, IgG2a, IgG1, and IgA titers with no statistical differences between them (Figure S2b). However, PEG OVA NPs induced strong anti-PEG total IgG, IgG1, and IgA responses (Figure S2c), indicating that PEGylated NPs may trigger unintended or allergic responses despite their positive anti-OVA titers. Several studies have reported allergic responses induced by PEG and cautioned against the use of PEG for the vaccine formulation as increased anti-PEG antibody levels induced by PEGylated mRNA vaccine were correlated with systemic reactogenicity.^24,58−61^ In contrast, significant antichitosan titers were not detected in mice immunized with LBL OVA NPs (Figure S 2d). This suggests that LBL modification of NPs using chitosan and CpG could be a better approach for IN vaccination to avoid off-target immune responses while retaining the benefits of high mucosal diffusivity and antigen immunogenicity. These results motivated the development and assessment of LBL NPs as flu vaccines. While HA-4M2e NPs are not used for the biodistribution study, OVA and HA-4M2e NPs share similar NP sizes and surface charges with and without LBL coatings. As reported in our previous study,^36^ the surface charge played the most critical role in the diffusivity of NPs in mucus, with the LBL coating significantly improving the mucus diffusivity of NPs compared to PEG coated or single-charged NPs.^36^ Therefore, we propose that LBL OVA and LBL HA-4M2e NPs would display a similar nasal delivery behavior.

### Development and Characterization of the Layer-by-Layer HA-4M2e Nanoparticle Vaccine

**Figure 2:**
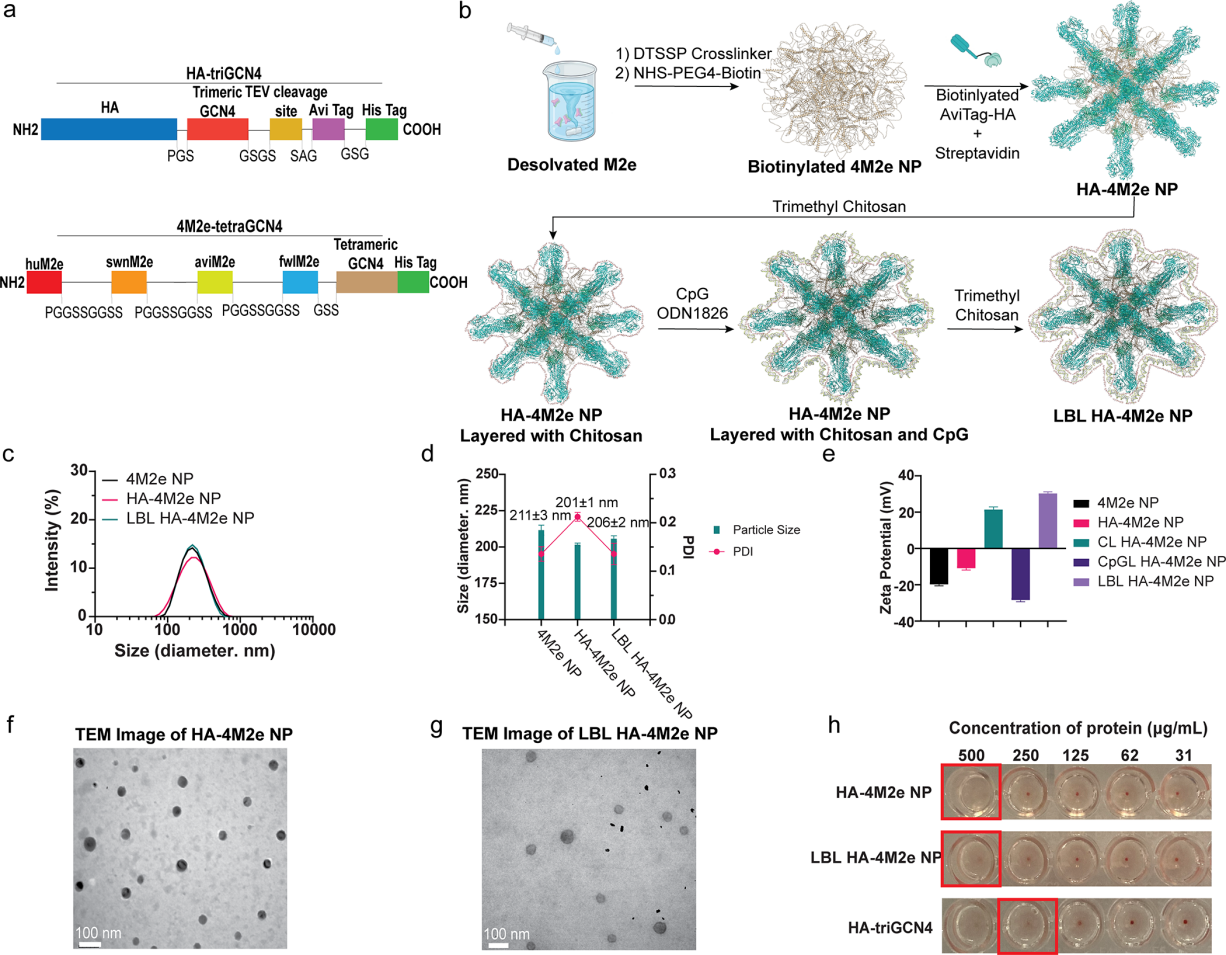
Characterization of LBL HA-4M2e NPs. (a) Diagram of HA-triGCN4 and 4M2e-tetraGCN4 proteins and their compositions. (b) Schematic illustration of LBL HA-4M2e NP synthesis. (c, d) Size distribution and hydrodynamic sizes (diameter) measured by dynamic light scattering (DLS). (e) Zeta potential measured by electrophoretic light scattering indicates the surface charges of NPs. (f, g) TEM images of HA-4M2e NPs and LBL HA-4M2e NPs. (h) Hemagglutination assay for the assessment of the functional structure of HA conjugated to 4M2e NPs. The data for nanoparticle size, PDI, and zeta potential were collected from three separate batches of NPs.

To assess the effect of LBL on IN influenza vaccine NPs, we selected M2e for the NP core and HA for the coating as our previous work demonstrated this combination to be effective for IM vaccination.^62^ However, in that design, HA was randomly bound to the M2e core with no control over orientation, as exists on native virus. Humoral immune responses can be significantly improved by optimizing the orientation of antigens presented on NPs,^63,64^ structure of antigens in NPs,^65^ and antigen valency.^66−68^ Biotin–streptavidin interaction with site-specific biotinylation of antigens using the BirA enzyme has been utilized for multivalent antigen presentation with controlled orientation without disrupting the native structure of antigen.^69^ Therefore, we chose this method to attach HA site-specifically to the M2e core to create a vaccine NP to evaluate the impact of the LBL coating for IN administration. The trimeric conformation of HA (H1N1 A/California/04/2009) was stabilized by fusion to the trimeric GCN4 coiled coil (HA-triGCN4),^70,71^ and an Avi-tag was fused to the C-terminus of triGCN4 for site-specific biotinylation to orient HA away from the NP surface (Figure 2a,b, Table S1). The M2e antigen consisted of a linear combination of human, swine, avian, and fowl M2e consensus sequences with tetrameric conformation induced by fusion to tetrameric GCN4 (4M2e-tetraGCN4) as previously described.^62^ HA-triGCN4 and 4M2e-tetraGCN4 were expressed by Expi239F and *E. coli* BL21*(DE3), respectively, and their expression was confirmed by SDS-PAGE and Western blot (Figure S3a). Transient transfection of Expi293F cells yielded only ∼58 μg of HA-triGCN4 per 50 mL of culture. To improve the yield of HA-triGCN4, stably transfected monoclonal Expi293F cells were selected from a pool of transiently transfected cells, expanded, and adapted to the suspension culture. Stable cells increased the expression level of HA-triGCN4 by ∼13-fold when harvested on day 4 (Figure S4e) at peak viable cell density (Figure S4c,d).

The resulting HA-triGCN4 and 4M2e-tetraGCN4 antigens exhibited trimeric and tetrameric structures, as shown in Figure S3b. Using the antigens, we synthesized NPs by desolvating 4M2e-tetraGCN4 with ethanol (4M2e NPs). 4M2e NPs were stabilized by the amine-reactive DTSSP cross-linker as shown in Figure 2b and biotinylated. To conjugate HA-triGCN4 onto the surface of 4M2e NPs (HA-4M2e NPs), the Avi-tag of HA-triGCN4 was biotinylated and bound to streptavidin, which, in turn, bound to biotinylated desolvated 4M2e-tetraGCN4 NPs (Figure 2b). LBL HA-4M2e NPs were generated with the same methods used for the generation of the OVA NPs to produce the chitosan–CpG–chitosan coating (Figure 2b).

4M2e NPs, HA-4M2e NPs, and LBL HA-4M2e NPs displayed similar monodisperse size distributions with hydrodynamic sizes of ∼200–215 nm as measured by dynamic light scattering (DLS) in Figure 2c,d. The size of HA-4M2e NPs (∼201 ± 1 nm) was slightly smaller than that of 4M2e NPs partly due to the loss of some core proteins during the sonication. However, after coating HA-4M2e NPs with chitosan and CpG ODN, the size of NPs slightly increased to 206 ± 2 nm, with all nanoparticle formulations under 0.25 for the PDI values, indicating a narrow size distribution.^72−74^ The surface charges of 4M2e NPs and HA-4M2e NPs were negative as indicated by their zeta potentials of −20 and −11 mV, respectively, indicating the successful conjugation of HA (Figure 2e). When HA-4M2e NPs were layered with cationic chitosan, the zeta potential changed to +21 mV, then to −28 mV with the anionic CpG layer, and finally to +30 mV with the outer chitosan layer (Figure 2e). The zeta potential after each layer correlated with the anionic or cationic polymer in the continuous phase, demonstrating the successful modification of the surface of the HA-4M2e NPs, and the high zeta potential >30 mV indicated the stabilization of the final nanoformulation via electrostatic repulsion.^75^ Western blot revealed that HA-4M2e NPs consist of approximately 20% HA-triGCN4 and 80% 4M2e-tetraGCN4 proteins by band intensity (Figure S3c). Transmission electron microscopy (TEM) showed that both HA-4M2e NPs and LBL HA-4M2e NPs formed spherical particles with dehydrated diameters ranging from ∼40 to ∼70 nm (Figure 2f,g). Hemagglutination activity was observed at 500 μg/mL of NPs for HA-4M2e NPs (Figure 2h), revealing that the conjugated HA-triGCN4 antigen was functionally folded for presentation to B cells. LBL HA-4M2e NPs also exhibited hemagglutination; however, chitosan can induce hemagglutination, so it is not necessarily showing that HA is accessible after LBL.^76^

### Intranasal Immunization with Layer-by-Layer HA-4M2e Nanoparticles Elicits Strong Humoral Immune Responses against HA and M2e

**Figure 3:**
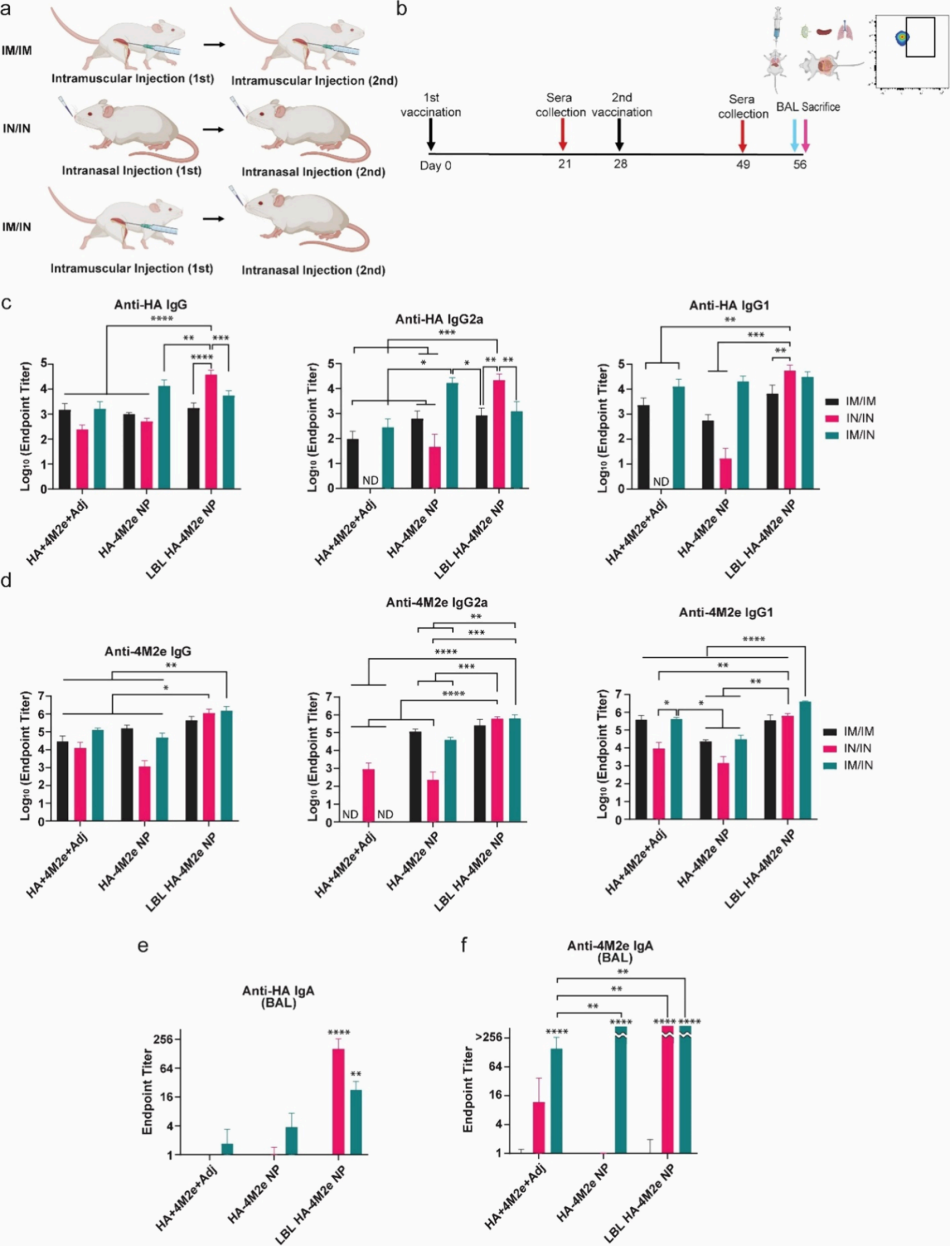
Systemic and local humoral immune responses against HA and 4M2e. (a) Different routes of vaccination: intramuscular priming and boost, intranasal priming and boost, and intramuscular priming followed by intranasal boost. (b) Schematic representation of the timeline for vaccination and collection of sera and BAL fluid. (c, d) Anti-HA (c) and anti-4M2e (d) IgG, IgG2a, and IgG1 titers in sera collected 49 days postpriming from mice immunized with HA + 4M2e + Adj, HA-4M2e NP, and LBL HA-4M2e NP. (e, f) Anti-HA (e) and anti-4M2e (f) IgA titers in BAL fluid collected 56 days postpriming from mice immunized. PBS-treated mice titers were below the limit of detection, so they are not shown. The *p* values (*n* = 5) were determined by two-way ANOVA with Tukey’s *post hoc* multiple comparison analysis: * for ≤0.05, ** for ≤0.01, *** for ≤0.001, and **** for ≤0.0001. ND represents not detectable values.

Given that LBL HA-4M2e NPs preserved the trimeric structure of HA, incorporated CpG, and chitosan adjuvants and enabled IN delivery to NALT and lungs, we hypothesized that LBL HA-4M2e NPs would induce a robust humoral immune response especially against HA. We examined the immunogenicity of LBL HA-4M2e NPs via different vaccination routes compared to uncoated HA-4M2e NPs and a soluble mixture of HA, 4M2e, and CpG and chitosan adjuvants (HA + 4M2e + Adj) to mimic commercially recombinant protein vaccines as adjuvants have been shown to enable detectable and improved antibody titers over soluble antigen alone.^77−79^ IgG2a and IgG1 titers were also measured to assess Th1 and Th2 responses, respectively, which are essential for the host defense directed against pathogens; the Th1 response is associated with cellular immune responses against intracellular virus, while the Th2 response promotes humoral immune responses and is important for immunity against extracellular pathogens.^80−83^ BALB/c mice were IM primed on day 0 and boosted on day 28 (IM/IM), IN primed and boosted (IN/IN), or IM primed and IN boosted (IM/IN) (Figure 3a,b). Few differences in humoral immune responses were seen prior to boosting (Figure S5a,b). Postboost titers demonstrated that LBL HA-4M2e NPs elicited strong anti-HA total IgG, IgG2a, and IgG1 responses via IN/IN immunization (Figure 3c). As LBL did not enhance titers for the IM/IM or IM/IN routes, it is clear that the coating strategy designed for IN delivery benefits IN vaccination. While HA-4M2e NPs did not substantially improve anti-HA total IgG, IgG2a, and IgG1 titers via IM/IM or IN/IN immunization over the soluble mixture + adjuvant, a high anti-HA IgG2a titer was observed in IM/IN immunized mice (Figure 3c). This is consistent with the benefits reported for mixed route administration.^84−86^ Consistent with an anti-HA humoral immune response, strong anti-4M2e IgG, IgG2a, and IgG1 responses were seen in mice vaccinated with LBL HA-4M2e NPs (Figure 3d). However, this was observed for both IN/IN and IM/IN routes. Similar to anti-HA responses, uncoated HA-4M2e NPs did induce significantly high anti-4M2e IgG2a titers by IM/IM or IM/IN compared to soluble adjuvanted antigens, which induced no IgG2a by these routes (Figure 3d) even though CpG ODN 1826 is an adjuvant that can promote Th1 immune responses. Soluble adjuvanted antigens generally tended to favor anti-HA and anti-4M2e IgG1 responses associated with Th2 (Figure 3c,d), especially for the IM/IM and IM/IN routes. These results show the value of the NP for antiviral immune responses in nonmucosal administration. Despite the weak Th1 immunogenicity induced by adjuvants in soluble antigens, the adjuvants significantly enhanced Th1 associated immunogenicity of antigens when layered onto LBL HA-4M2e NPs. Therefore, a unique combination of muco-penetrating properties and adjuvant effects of chitosan and CpG layers on NPs may combine to improve the effectiveness of intranasal vaccination.

The benefit of both NPs and LBL coating is the most obvious in the IgA titers from the bronchoalveolar lavage (BAL) fluid. Similar to anti-HA IgG, the highest anti-HA IgA titer was from IN/IN LBL HA-4M2e NP immunized mice (Figure 3e), while both IN/IN and IM/IN immunization with LBL-4M2e NPs elicited strong anti-4M2e IgA responses (Figure 3f). IM/IN HA-4M2e NPs elicited a robust anti-4M2e IgA response but not anti-HA IgA. This implies that antigen-specific immune responses may be dependent on the routes of vaccination and/or the location of the antigen on the NP. It is well-known that different routes of administration affect the quality of immune responses as antigens are transported to different sites such as lymph nodes,^87−90^ and the location of antigens in the NP core or on the surface could also impact transport and accessibility. HA exposed on the NP surface could interact with sialic acid receptors on respiratory epithelial cells, a primary target of HA of influenza.^91^ It is notable that soluble adjuvanted antigens produced negligible anti-HA IgG2a and IgG1 titers via the IN/IN route, while the anti-4M2e IgG2a titer was substantially higher with IN/IN immunization compared to IM/IM and IM/IN, which contrasts with the immune responses observed with LBL HA-4M2e NPs. This implies that the observed antigen-specific immune responses could have been affected by the location of antigens on the NP, not only their identity. However, the correlation between administration routes and immune responses elicited by different antigens such as HA and M2e has not been extensively reported, and the associated mechanism remains unclear. In concert, the results generally showed that LBL HA-4M2e NPs markedly enhanced both anti-HA and anti-4M2e humoral immune responses, especially when they were intranasally administered to mice, the route for which they were designed.

### Immunization of Mice with LBL HA-4M2e Nanoparticles Elicits Strong T-Cell Immune Responses against HA and M2e

**Figure 4:**
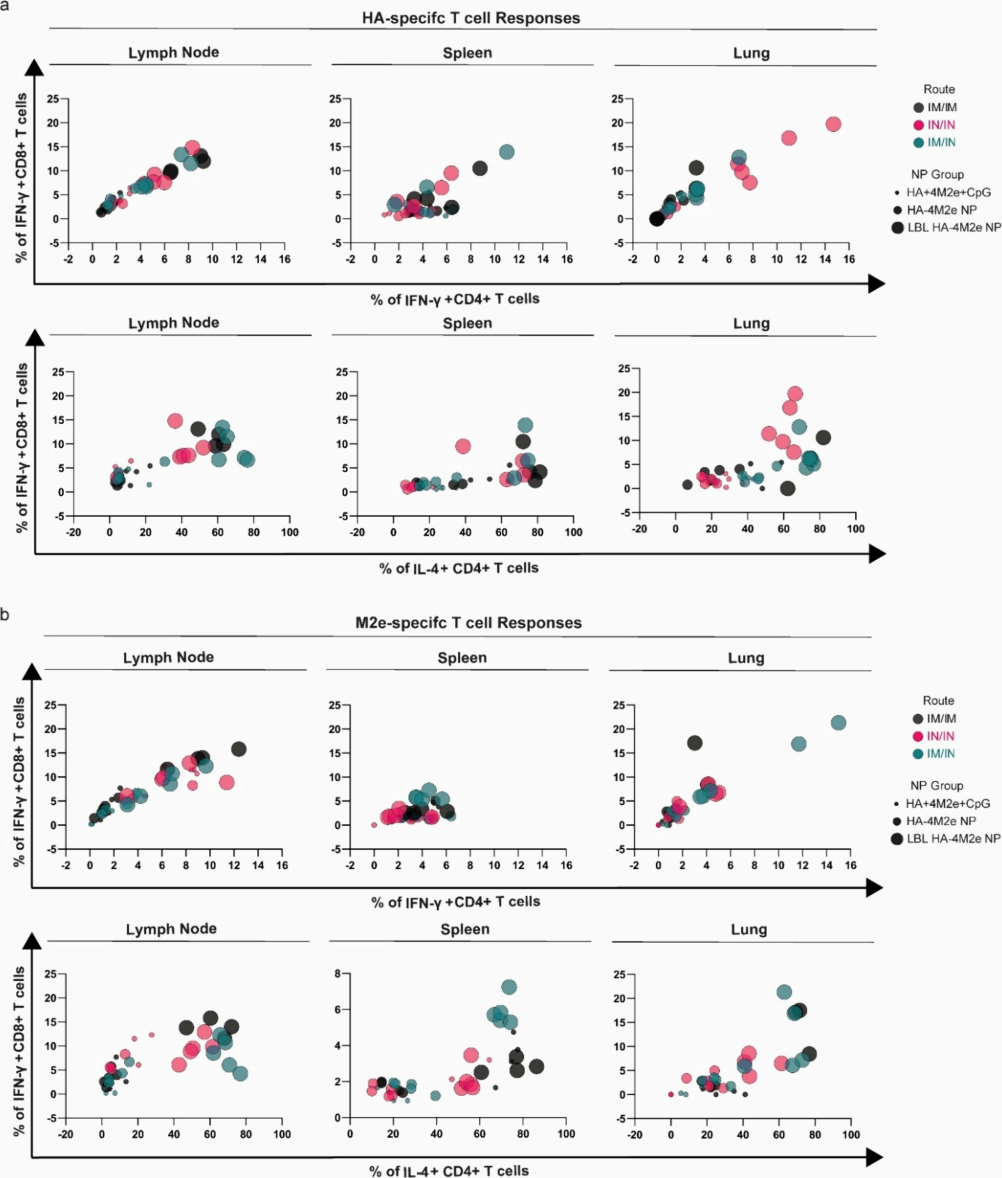
Antigen-specific cellular immune responses at 8 weeks post prime. (a) Percent populations of restimulated HA-specific IFN-γ^+^ CD8^+^ T cells, IFN-γ^+^ CD4^+^ T cells, and IL-4^+^ CD4^+^ T cells in the lymph node, spleen, and lung. (b) Percent populations of restimulated M2e-specific IFN-γ^+^ CD8^+^ T cells, IFN-γ^+^ CD4^+^ T cells, and IL-4^+^ CD4^+^ T cells. Comparison of the percentage of T-cell populations generally shows potent HA- and M2e-specific cellular immune responses induced by LBL HA-4M2e NPs especially when mice were administered via IN/IN and IM/IM or IM/IN routes, respectively. Bar graphs of the same data showing statistical analysis are shown in Figure S7.

We next evaluated cellular immune responses by harvesting lymph nodes, spleens, and lungs from immunized mice 56 days postprime and performing intracellular cytokine staining (ICS) on T cells for analysis by flow cytometry (Figure S6). LBL HA-4M2e NPs significantly increased populations of HA-specific IFN-γ^+^ CD8^+^ T cells (Figure 4a and Figure S7a), IFN-γ^+^ CD4^+^ T cells (Figure 4a and Figure S7b), and IL-4^+^ CD4^+^ T cells (Figure 4a and Figure S7c) in the lymph nodes and lungs over uncoated NPs and soluble mixture + adjuvant formulation. For lymph node T cells, activation is independent of the route of administration, but for lungs, IN/IN delivery gives a stronger response for most cell types. LBL HA-4M2e NPs also induced HA-specific IFN-γ^+^ CD8^+^ and IL-4^+^ CD4^+^ T cells in the spleen (Figure 4a and FigureS7a,c). Consistent with anti-HA humoral immune responses, IN/IN immunization with LBL HA-4M2e NPs elicited potent anti-HA IFN-γ^+^ CD4^+^ T (Th1) cell-mediated immune responses in the lungs, whereas pronounced anti-HA Th1 immune responses were not detected in mice administered with the soluble mixture + adjuvant formulation or HA-4M2e NPs (Figure 4a and Figure S7a,b). Strong Th2 responses were also seen in mice immunized with LBL HA-4M2e NPs, especially by IM/IM or IM/IN (Figure 4a and Figure S7c). In alignment with anti-4M2e humoral immune responses, strong anti-M2e Th1 responses were established in mice IN/IN immunized with the soluble mixture + adjuvant formulation compared to IM/IM and IM/IN routes. These results suggest that IN/IN immunization with an adjuvanted vaccine tends to induce Th1-biased immune responses.

M2e-specific T-cell activation trends were very similar to HA-specific T-cell activation in that LBL NPs induced larger percentages of all T-cell types measured especially in the lymph nodes and lungs (Figure 4b and Figure S7d–f); however, the dependence on the administration route was different. In the lymph nodes, LBL HA-4M2e NPs activated the highest percent populations of M2e-specific IFN-γ^+^ CD8^+^ T cells and IFN-γ^+^ CD4^+^ T cells by IM/IM immunization (Figure 4b and Figure S7d,e). In contrast to HA-specific T-cell immunity, where IN/IN was the best, IM/IN immunized mice exhibited the highest M2e-specific IFN-γ^+^ CD8^+^ and IFN-γ^+^ CD4^+^ T-cell populations in the lungs (Figure 4b and Figure S7d,e). These observations are aligned with the trends observed from anti-HA and anti-4M2e humoral immune responses, where IN/IN immunization with LBL HA-4M2e NPs strongly induced anti-HA humoral immunity while IM/IN and IN/IN immunization significantly enhanced anti-4M2e humoral immunity (Figure 3). This shows that there is a unique anti-HA immune response elicited by IN/IN LBL HA-4M2e NPs. Taken together, these data suggest that LBL HA-4M2e NPs promoted strong dual antigen T-cell activation superior to the soluble combination of antigens and LBL components as well as unmodified NPs. Since IN/IN vaccination with LBL HA-4M2e NPs consistently induces the strongest humoral and cellular immune responses, we primarily focused on the IN/IN route to examine the effectiveness of LBL HA-4M2e NPs as an intranasal vaccine for the remainder of experiments, with IM/IM as the control.

### Intranasal Immunization of Mice with LBL HA-4M2e Nanoparticles Elicits Long Lasting Humoral Responses against HA and M2e

**Figure 5:**
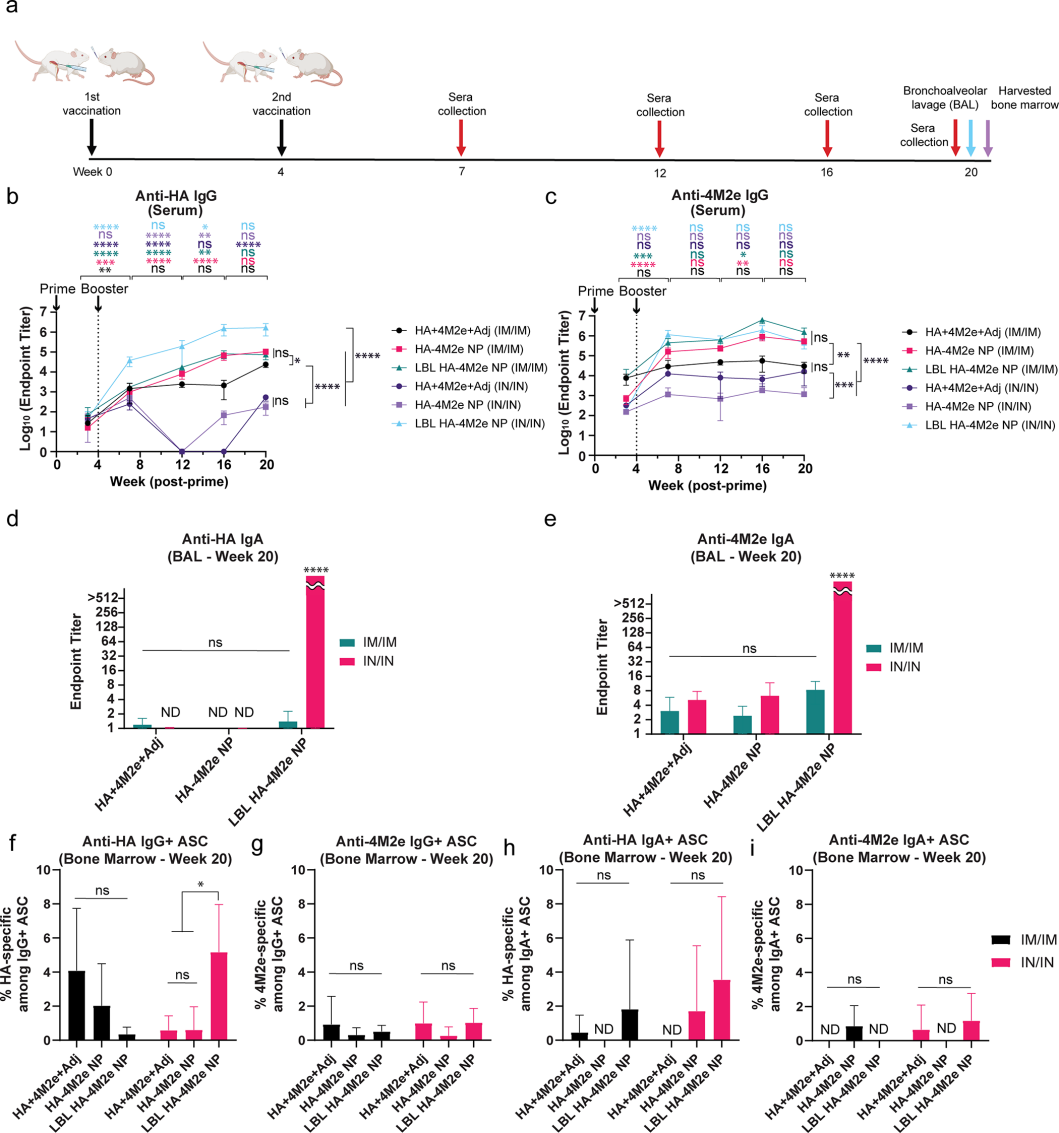
Persistence of humoral immune responses against HA and 4M2e. (a) Timeline for vaccination and collection of sera, BAL fluid, and bone marrow. (b, c) Anti-HA (b) and anti-4M2e (c) IgG titers in sera collected on weeks 3, 7, 12, 16, and 20 postprime from mice immunized IM/IM and IN/IN with HA + 4M2e + Adj, HA-4M2e NP, and LBL HA-4M2e NP. (d, e) Anti-HA (d) and anti-4M2e (e) IgA titers in BAL fluid collected on 140 days postprime. (f–i) Quantification of HA- and 4M2e-specific IgG (f, g) and IgA (h, i) secreting long-lived bone marrow B cells after stimulating with R848/IL-2 (B-Poly-S reagent). PBS-treated mice titers were below the limit of detection and are not shown. ND stands for not detectable values. Comparisons across groups are only shown for week 20 time point. The *p* values (*n* = 5) were determined by two-way ANOVA with Tukey’s *post hoc* multiple comparison analysis: ns for not significant, * for ≤0.05, ** for ≤0.01, *** for ≤0.001, and **** for ≤0.0001.

To assess the duration of IgG and IgA responses, given the reported low durability of IN vaccines,^15,16^ serum antibodies were collected from immunized mice 8, 12, and 16 weeks after boost (Figure 5a). High anti-HA and anti-4M2e IgG titers were seen in mice immunized IM/IM with HA-4M2e NPs or LBL HA-4M2e NPs out to 20 weeks (Figure 5b,c). At week 20, HA-4M2e NPs and LBL HA-4M2e NPs via the IM/IM route induced higher anti-HA (Figure 5b) and anti-4M2e IgG titers (Figure 5c), respectively, compared to HA + 4M2e + Adj, while mice IM/IM immunized with LBL HA-4M2e NPs showed a decrease in anti-M2e IgG levels from week 16 to week 20. The comparison between HA + 4M2e + Adj and HA-4M2e NP suggests that HA-4M2e NPs promote a relatively more durable humoral immune response than HA + 4M2e + Adj (Figure 5b,c). In contrast, IN/IN vaccination revealed a clear long-term benefit for anti-HA and anti-M2e IgG titers from LBL HA-4M2e NPs, which were higher than for IM/IM administration (Figure 5b,c). Anti-HA IgG titers induced by IN/IN LBL HA-4M2e NPs increased over time, while the other groups exhibited a large drop in systemic humoral immune responses at weeks 12 and 16 (Figure 5b). All IN/IN formulations maintained anti-M2e IgG levels over 20 weeks, but LBL HA-4M2e NP titers were significantly higher than those of HA + 4M2e + Adj and HA-4M2e NPs at week 20 (Figure 5c). These findings indicate that a combination of both NP and LBL formulations with chitosan and CpG is necessary for inducing persistently high anti-HA and M2e systemic IgG titers when delivered intranasally. Importantly, mice vaccinated IN/IN with LBL HA-4M2e NPs were the only group that showed high anti-HA and anti-4M2e IgA titers collected via bronchoalveolar washing at week 20, demonstrating the efficacy of LBL formulation of NPs for potent and durable mucosal IgA response in the respiratory tract (Figure 5d,e). While other cationic and anionic molecules may be used for LBL to enhance mucosal diffusion, the use of adjuvant polyelectrolytes provides an additional advantage of immune stimulation. It has been previously reported that significantly high anti-HA stalk and anticonsensus M2 (sM2) serum IgG and mucosal lung IgA titers at ∼ week 26 postimmunization were only seen in mice that were immunized IN (three vaccine doses: IN on weeks 0, 2, and 4) with chitosan formulated influenza vaccine NPs prepared by simple ionic interactions between poly-gamma-glutamate, chitosan, and recombinant sM2 from the ectodomain and cytoplasmic domain fused to the HA stalk peptide (sM2HA2) and cholera toxin subunit A1 (CTA1).^92^ However, without CTA1, immune responses induced by the NPs were comparable to a soluble mixture of sM2HA2 with and without CTA1. In another study, it was shown that a combination of inactivated influenza virus and CpG encapsulated in chitosan nanospheres induced the highest serum IgG and IgA (in nasal wash) titers in rabbits against H1N1 influenza virus at week ∼13 postintranasal priming (four vaccine doses: IN at weeks 0, ∼6, and ∼9 and IM at week ∼11) in comparison with inactivated virus incorporated in chitosan nanospheres, inactivated virus and CpG in suspension, and inactivated virus and *Quillaja* saponin (QS) adjuvants encapsulated in chitosan nanospheres.^93^

In addition to durable antibody titers, the memory B-cell response defines the quality of long-lasting vaccine-induced humoral immunity as memory B cells are long-lasting and can be rapidly activated upon re-exposure to antigens.^66,94−96^ It is therefore important to establish a memory B-cell response for long-term immunity. To delineate the memory B-cell response, the surface of cells harvested from lymph nodes was first stained with anti-B220/CD45R, anti-CD273, and anti-CD73 antibodies and gated to identify CD273^+^ CD73^+^ IgD^–^ B cells (Figures S8a and Figure S9). There were no differences in the percent population of total memory B cells among vaccine groups (Figures S8b and Figure S9). However, antigen-specific B-ELISpot conducted using bone marrow cells from vaccinated mice restimulated with R848 and IL-2 revealed that intranasal vaccination with LBL HA-4M2e NPs improved the HA-specific IgG^+^ long-lived plasma cell response compared to the other formulations (Figure 5f,g). Consistently, anti-HA IgG titers secreted from bone marrow cells immunized with LBL HA-4M2e NPs were the highest (Figure S10a,e). These results are in line with the potent systemic anti-HA humoral immune responses seen in mice immunized IN with LBL HA-4M2e NPs (Figure 5b). Nevertheless, there was no strong correlation observed between IgA titers (Figure 5d) and restimulated IgA antibody-secreting cells (ASCs) in the bone marrow (Figure 5h). With regard to anti-4M2e ASCs, we did not observe any statistically significant secretion improvement from bone marrow B cells (Figure 5g,i). Although LBL HA-4M2e NPs statistically enhanced anti-HA IgA and anti-4M2e IgG and IgA titers secreted from bone marrow cells at week 20 (Figure S10b–h), the increases in the titers were not as pronounced as for anti-HA IgG. Altogether, these observations indicate that IN immunization with LBL HA + 4M2e NPs established an enhanced anti-HA IgG^+^ long-lived plasma cell response but did not significantly improve IgA^+^ long-lived plasma cell response or anti-4M2e long-lived plasma cell response in the bone marrow. The bone marrow serves as a survival niche for long-lived plasma cells generated in systemic immune responses,^94,97,98^ and these long-lived plasma cells can survive for many years and provide long-term humoral protection. Although whether mucosal plasma cells can migrate to the bone marrow for long-term humoral immunity has remained a subject of continuing debate and uncertainty,^99,100^ a study hinted that mucosal plasmablasts can become long-lived bone marrow plasma cells.^101^ However, we did not detect notably increased IgA^+^ long-lived bone marrow plasma cells after intranasal vaccination, implying that durable mucosal humoral immune responses (Figure 5h,i) were mainly provided from NALT. Overall, LBL HA-4M2e NPs significantly improved humoral immune responses, especially durable mucosal IgA titers and HA-specific IgG^+^ long-lived bone marrow plasma cells.

### Intranasal Vaccination with LBL HA-4M2e NPs Enhances Prophylactic Effectiveness against Influenza Virus Challenge and Significantly Lowers Lung Virus Titers

**Figure 6:**
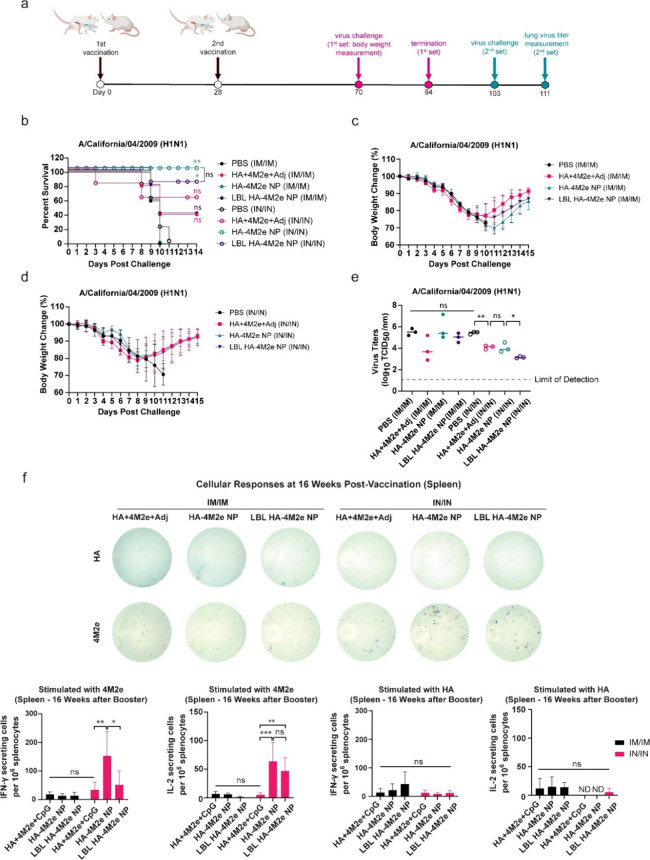
Immune protection conferred against challenges with H1N1 and pdm09 influenza A viruses in mice. (a) Timeline for intramuscular and intranasal vaccination and lethal virus challenge. (b–d) Survival rate (b) and body weight (c, d) of mice (*n* = 5) that were challenged with 4× LD_50_ H1N1 influenza A virus (A/California/04/2009) 6 weeks after the second vaccination. (e) Lung viral titers (*n* = 5) were measured from vaccinated mice after being challenged with 3× LD_50_ H1N1 influenza A virus 10.5 weeks after the second vaccination. The dashed line is the limit of detection. (f) M2e- and HA-specific IFN-γ (blue spots) and IL-2 (red spots) secreting splenocytes were quantified by T-ELISpot at 16 weeks after a booster. ND stands for not detectable values. Survival analysis was performed by Kaplan–Meier analysis with the log rank test: ns for not significant, * for ≤0.033, and ** for ≤0.002 compared to PBS control group. The *p* values (*n* = 5) were determined by two-way ANOVA with Tukey’s *post hoc* multiple comparison analysis: ns for not significant, * for ≤0.05, ** for ≤0.01, *** for ≤0.001, and **** for ≤0.0001.

Licensed intramuscular influenza vaccines were recommended over FluMist, the main licensed intranasal influenza vaccine, for protection against influenza A(H1N1)pdm09 from 2016 to 2018 due to the lower effectiveness of the intranasal vaccine.^11^ To evaluate the prophylactic effectiveness of LBL HA-4M2e NPs as an intranasal influenza vaccine compared to different vaccine formulations and the intramuscular route of administration, we challenged immunized mice with 4× the 50% lethal dose (LD_50_) of H1N1 influenza (A/California/04/2009) 6 weeks post boost immunization (Figure 6a). Mice with body weight loss greater than 25% were euthanized. Most mice with IM vaccination were not protected against lethal virus challenge, except for the soluble HA + 4M2e + Adj IM/IM group, which showed 40% partial survival protection (Figure 6b). In contrast, higher survival rates and less body weight losses were seen in mice immunized IN with HA-4M2e NP, LBL HA-4M2e NP, and HA + 4M2e + Adj, whose survival rates were 100, 80, and 60%, respectively (Figure 6b–d). These results show that IN immunization can be an effective route for protection with NP vaccine formulations. The challenge was repeated at 10.5 weeks after the boost to better compare the protective efficacy of different vaccine formulations and routes by assessing lung viral titers. Consistent with their strong immunogenicity, IN immunization with LBL HA-4M2e NPs most significantly decreased lung viral titers in challenged mice (Figure 6e) by ∼229-, 9-, and 8-fold compared to the naive, HA + 4M2e + Adj, and HA-4M2e NP IN/IN groups, respectively. The IN/IN HA-4M2e NP group exhibited a moderate reduction of 6-fold in lung viral titers compared to the IM/IM HA-4M2e NP group despite its ∼30-fold decrease in lung viral titers from the naive group. It is notable that soluble HA + 4M2e + Adj vaccination showed similar lung viral titers between IN/IN and IM/IM delivery. The impact of IN delivery was found to be the most prominent in the LBL HA-4M2e NP group, which displayed 70-fold lower lung viral loads by the IN/IN route compared to IM/IM vaccination (Figure 6e).

We speculated that IN/IN vaccination with NPs could have also induced T-cell immune responses, which could have contributed to the enhanced protective effectiveness. To examine durable T-cell responses, we performed an ELISpot assay for the quantification of M2e- and HA-specific T cells harvested from mice at 16 weeks postboost vaccination. The data revealed that a substantially high number of M2e-specific IFN-γ secreting splenocytes appeared in mice IN vaccinated with HA-4M2e NPs (Figure 6f), although HA-specific IFN-γ cellular responses were not robust. In addition, IN vaccination with both HA-4M2e NPs and LBL HA-4M2e NPs improved long-term M2e-specific IL-2^+^ T-cell responses, which are known to promote the activation and growth of T cells and appear to play critical roles in T-cell fates including memory T-cell responses.^102−105^ This suggests that durable M2e-specific T-cell responses were established in the HA-4M2e NP vaccine group and, to a lesser extent, the LBL HA-4M2e NP group, which had a pronounced effect on survival rates upon lethal influenza challenge. Furthermore, these results indicate that IN vaccination using NPs can be an effective route to promoting durable T-cell responses. Interestingly, although anti-HA humoral immune responses persisted at least to week 20, HA-responsive T cells did not last long (Figure 6f), implying that durable T-cell responses are also dependent on antigens. Together, HA in NPs enhanced long-term humoral immune responses, while M2e significantly contributed to durable cellular immune responses, revealing site-specific conjugation of HA onto M2e NPs to be an effective strategy to promote both durable humoral and cellular immune responses.

## Conclusions

In this work, we report the development of LBL HA-4M2e NPs as a vaccine platform for effective IN vaccination against influenza. Chimeric tetramer M2e antigen NPs were site-specifically conjugated to HA antigen and then coated with alternating layers of cationic chitosan and anionic CpG adjuvants to enable delivery across the nasal mucosa to NALT. We observed strong anti-HA and anti-M2e systemic IgG and mucosal IgA titers from mice IN immunized with LBL HA-4M2e NPs both shortly after vaccination and 5 months after the prime. The IM/IN route was also effective for anti-M2e production. Consistently, IN/IN immunization with LBL HA-4M2e NPs promoted HA-specific T-cell responses, especially in the lungs, which is critical for influenza protection, while the highest percent populations of M2e-specific T cells were seen in mice immunized by IM/IM and IM/IN LBL HA-4M2e NPs. IM/IN “prime and pull” immunization has been reported as an effective strategy for eliciting robust immunity, particularly in augmenting lung-resident T-cell responses.^85,106−108^ In our study, however, IN/IN immunization using LBL HA-4M2e NPs generally induced potent HA-specific immunity, including both high IgA titers and strong T-cell responses in the lungs. Additionally, IM/IN prime and pull regimens enhanced M2e-specific immunity. Previous studies have shown that prime and pull immunization with influenza vaccines formulated with nucleoprotein effectively augmented lung T-cell responses.^106,107^ In contrast, virus-like particles (VLPs) presenting HA induced comparable immune responses between IM/IM and IM/IN immunizations.^109^ The observation of a distinct trend for antigen-specific immune responses dictated by different routes of vaccination should be further studied both to understand if the reasons are immunological or spatial and to guide vaccination route selection for future multiantigen vaccines. It should also be noted that the combinations of vaccine modalities and different vaccination routes significantly affect immune responses,^109,110^ making it more challenging to study the effects of vaccination on immune responses.

The potent immune responses induced by IN immunization with LBL HA-4M2e NPs were accompanied by the lowest virus titers in the lungs of mice after the lethal influenza challenge. It is noteworthy that durable M2e-responsive T cells were observed in mice immunized with IN with HA-4M2e NPs without adjuvants. The long-lasting M2e-specfic T-cell responses established in the HA-4M2e NP group may provide a potential explanation for the 100% survival protection against influenza challenge. Building on this line of reasoning, LBL assembly may not be required for complete protection against virus challenges in this study. However, it is important to note that the virus challenge in this study was performed 6 weeks postvaccination, whereas virus challenges are typically conducted in mice within 2–4 weeks postvaccination. Over this extended time frame, T cells might have undergone significant contraction by 5 to 6 weeks postvaccination. This is partially supported by the long-term immune response measurements, where T-cell responses induced by IN LBL HA-4M2e NPs were weaker at week 16 postvaccination compared to those induced by IN HA-4M2e NPs. This reduction could be attributed in part to the T-cell exhaustion due to the strong early T-cell activation^111−113^ at 8 weeks by IN LBL HA-4M2e compared to uncoated NP, or to the biased T-cell differentiation driven by robust IL-4^+^ CD4^+^ T-cell responses in the IN LBL HA-4M2e NP group. Despite this, IN LBL HA-4M2e NPs induced the most durable humoral immune responses, resulting in the lowest virus titers in the IN LBL HA-4M2e NP group. This suggests that LBL coating can be an effective strategy for neutralizing viruses in lungs. Furthermore, if LBL NPs are formulated with antigens or components capable of augmenting memory Th1 responses, then LBL coating could serve as a promising approach for the development of IN vaccines, and its application to other antigens and vaccine NPs should be evaluated in depth.

The limitation of this study includes a lack of direct comparison to licensed whole-pathogen influenza vaccines or recombinant influenza vaccines. However, it is notable that the intranasal administration of HA-4M2e NPs, especially with LBL coatings, significantly enhanced immunity against influenza compared to a mixture of recombinant HA and 4M2e antigens with CpG and chitosan adjuvants. Commercially available recombinant influenza vaccines such as Flublok are more effective than standard-dose unadjuvanted influenza vaccines when 3× HA antigen content of standard-dose flu vaccines is provided.^114,115^ Therefore, our results imply that LBL coatings can be an effective strategy for the formulation of an intranasal vaccine but should be tested against licensed influenza vaccines in future work.

In this work, IN vaccination notably enhanced immune responses and prophylactic effectiveness, rendering it a promising vaccination strategy for NPs against influenza. NPs fabricated from 4M2e and HA via biotin–streptavidin conjugation are an integral component of the influenza vaccine formulation, demonstrating the importance of antigen format. A recent study by Kastenschmidt et al. also showed how different antigen formats elicited distinct influenza-specific immune responses within the mucosal site using a human tonsil organoid, providing important implications for IN influenza vaccine development.^116^ While IN vaccination can significantly enhance both mucosal and systemic immunity, the duration of immune responses is often insufficient due to the low antigen uptake resulting from nasal delivery barriers.^15,16^ To address the unique physiological and chemical nature of the nasal cavity, we designed vaccine NPs with chitosan and CpG adjuvant LBL coatings to improve mucus diffusion^36^ and prolong antigen presentation. This approach significantly improved the durability of both HA- and 4M2e-specific IgG and IgA responses and M2e IL-2^+^ T-cell responses. Taken together, LBL coating of charged polymer adjuvants has significant benefit for enhancing the intranasal delivery and the durable systemic and mucosal immunogenicity of influenza protein NPs, and this approach could be applied to a wide range of NP types for different respiratory vaccines.

## Experimental Section

### OVA Protein Nanoparticle Fabrication

The fabrication of the OVA-NPs was performed using the desolvation method. One hundred microliters of a protein solvent phase that contained 10 mg/mL Ovalbumin EndoFit (Invivogen) in cell culture phosphate buffered saline (PBS) was mixed at 750 rpm, while a desolvent phase of 100% ethanol was added dropwise at a ratio of 1:4 by volume. Three batches were pooled together, and NPs were isolated using centrifugation at 21,000*g* for 10 min at 10 °C and resuspended by sonication in 400 μL PBS. 3,3′-Dithiobis(sulfosuccinimidyl propionate) (DTSSP, Thermo Fisher Scientific) was added at a molar ratio of 1:2.2 DTSSP/lysine residues to the NP suspension at 600 rpm at room temperature for 1 h. The solution was then centrifuged for 32 min at 21,000*g* at 4 °C. The supernatant was removed, and the pellet was resuspended in 500 μL PBS. NPs were sonicated on ice for 1 min, with 1 s on and 3 s off at 50% amplitude. NPs were centrifuged for 5 min at 2000*g* to remove any large aggregates, and the supernatant was collected as OVA NP.

For surface modification with PEG, OVA NPs (5 mg/mL) were buffer exchanged to sodium bicarbonate buffer (1 M, pH 8.3) through centrifugation at 18,000*g*. Afterward, 2.0 mg of *O*-[(*N*-succinimidyl)succinyl-aminoethyl]-*O*′-methylpolyethylene glycol with average Mn 750 (Thermo Fisher Scientific) was added and reacted at room temperature for 2 h. PEG-OVA NPs were collected using a 10 kDa molecular weight cutoff Amicon Ultra-4 Centrifugal Filter Unit (Millipore Sigma) to remove unreacted PEG.

For LBL surface modification, OVA NPs were prepared using a modified desolvation method from the above to adjust the size so that LBL and unmodified NPs had the same final diameter for animal experiments. Ovalbumin EndoFit (20 mg/mL, Invivogen) in 100 μL of PBS was desolvated under constant stirring at 700 rpm with 400 μL of 50:50 methanol/ethanol added dropwise. NPs were collected and cross-linked as above. After centrifugation at 10,000*g* at 4 °C, the supernatant was collected as the cores for LBL OVA NPs. A 1% w/v solution of low-molecular-weight trimethyl chitosan (Millipore Sigma) was prepared in endotoxin-free water (G-Biosciences). NPs were incubated in the chitosan solution for 10 min and briefly sonicated for a homogeneous coating. The suspension was then centrifuged at 18,000*g* at 4 °C, washed with endotoxin-free water, and centrifuged to remove excess chitosan twice. NPs were then added to a solution of 22.1 nM ODN 1826 VacciGrade (Invivogen) and briefly sonicated for a homogeneous coating of the anionic layer. NPs were then centrifuged at 18,000*g* at 4 °C and washed with endotoxin-free water. An additional coating of chitosan was added following the same procedure as the first layer. Then, LBL OVA NPs were centrifuged and resuspended in PBS for excess polyelectrolyte removal.

For the IN biodistribution study, fluorescent OVA, PEG OVA NPs, and LBL OVA NPs were synthesized by conjugating 15 wt % of OVA with Alexa Fluor 647 (Thermo Fisher Scientific) to yield a degree of labeling of ∼1.0.

### Nanoparticle Characterization

NP concentrations were determined using a BCA assay following the manufacturer’s protocol (Thermo Fisher Scientific). CpG ODN 1826 was quantified using a Qubit ssDNA Assay Kit (Thermo Fisher Scientific), and trimethyl chitosan was quantified using a Periodic Acid-Schiff (PAS) assay (Sigma-Aldrich). NP size distribution and zeta potential were assessed by dynamic light scattering (DLS) and electrophoretic light scattering (ELS) in 1× and 0.1× PBS, respectively, with a Malvern Zetasizer Nano ZS90 (Malvern Instruments, Westborough, MA). Measurements were carried out in triplicate with three distinct batches of particles. Each measurement consisted of 12–30 runs. Electrophoretic mobility was converted to zeta potential using the Smoluchowski approximation.

TEM imaging was performed to assess the physical sizes and shapes of NPs. Five microliters of NPs was dropped on a 300-mesh carbon film supported copper grid (Millipore Sigma) for 5 min. After the copper grid was washed with deionized water, the sample was stained with 5 μL of 1% phosphotungstic acid solution for 15 s and washed again with deionized water. The sample was then allowed to dry overnight and imaged at 80 kV using a JEOL 100 CX-II TEM. The content of dual antigen NPs (HA-4M2e NPs) was evaluated by Western blot with band intensity analysis using ImageJ.

### HEK-293 mTLR9 Cell Culture

The HEK-Blue mTLR9 (immortalized human embryonic kidney cells) cell line was obtained from Invivogen (cat. #: hkb-mtlr9) and cultured in Dulbecco’s modified Eagle’s medium (Corning) supplemented with 4.5 g/L glucose, 10% (v/v) fetal bovine serum (FBS), 100 U/mL penicillin, 100 μg/mL streptomycin (Amresco), 100 μg/mL Normocin (Invivogen), and 2 mM l-glutamine. Cells were incubated under humidified conditions at 37 °C and 5% CO_2_. After two passages, cells were maintained with the growth medium supplemented with 10 μg/mL of Blasticidin (Invivogen) and 100 μg/mL of Zeocin (Invivogen). Cells were passaged when a 70–80% confluency was reached.

### TLR9 Stimulation and Detection

Twenty microliters of LBL OVA NPs with varying concentrations and soluble CpG ODN 1826 as a control were added onto a flat-bottom 96-well plate. HEK-Blue mTLR9 cells at 80% confluence were washed with warm PBS and detached with Nunc Cell Scrapers (Thermo Fisher Scientific). A total of 80,000 cells were added to each well and incubated under humidified conditions at 37 °C in 5% CO_2_ for 24 h. Samples were read at 620 nm with a Synergy HTX Multimode Reader (Agilent).

### Protein Expression and Development of a Stable Cell Line

The pcDNA3.1 plasmid encoding HA-triGCN4 with Avi-tag was obtained from Gene Universal. A 6× His tag was introduced at the C-terminus for purification via Ni-NTA agarose affinity chromatography. Expi293F cells were transiently transfected by using an Expi293 Expression System kit (cat. #: A14635, Thermo Fisher Scientific) according to manufacturer’s instructions. Transfected Expi293F cells were grown in the Expi293 Expression Medium (Thermo Fisher Scientific) in baffled polycarbonate vented Erlenmeyer flasks (Thermo Fisher Scientific) at 37 °C 8% CO_2_ on a shaker set to 125 rpm for 5 days with the addition of the transfection enhancer 20 h post-transfection. The cells were then harvested and lysed in the lysis buffer containing 20 mM imidazole, 300 mM NaCl, and 50 mM NaH_2_PO_4_ by sonication 5 days post-transfection.

To develop a stable cell line expressing HA-GCN4, Expi293F cells were seeded in a flat-bottom six-well plate (Thermo Fisher Scientific) at 0.7 × 10^6^ cells/well with 1.5 mL of OptiMEM and transfected with a DNA mixture containing 3 μg of DNA, 380 μL of Opti-MEM (Thermo Fisher Scientific), 6 μL of Lipofectamine (Thermo Fisher Scientific), and 6 μL of the p3000 reagent (Thermo Fisher Scientific). Following transfection, Expi293F cells were incubated in Dulbecco’s modified Eagle’s medium (DMEM, Corning) supplemented with 10% fetal bovine serum (FBS, Avantor) 6 h post-transfection. After 48 h incubation at 37 °C and 8% CO_2_, the medium was replaced with DMEM supplemented with 10% FBS and Geneticin (G418 Sulfate, Thermo Fisher Scientific) at a final concentration of 300 μg/mL. The concentration of Geneticin was determined by generating a killing curve (Figure S1a); nontransfected Expi293F cells were incubated with Geneticin at concentrations ranging from 0 to 1000 μg/mL. The medium was replenished every 3 days until 80–90% confluency was achieved. Afterward, stable monoclonal cells were selected by performing a limiting dilution approach. Briefly, transfected cells were seeded on a flat-bottom 96-well plate (Thermo Fisher Scientific) at 0.8 cell/well in DMEM with 10% FBS and Geneticin. The medium was replenished every 3 days until 70% confluency was shown, and cells from wells with a single colony were selected and expanded on a flat-bottom six-well plate. To select monoclonal cells with a high level of expression, 50% of cells from each were harvested and lysed to extract HA-triGCN4 using 8 M urea buffer at pH 8.0. The level of expression was determined by Western blot analysis of the cell lysate (Figure S1b). The selected monoclonal stable cells were expanded and adapted to suspension growth in baffled polycarbonate vented Erlenmeyer flasks in the Expi293 Expression Medium. The stable cells were cultured for 4 or 5 days and lysed in a lysis buffer containing 20 mM imidazole, 300 mM NaCl, and 50 mM NaH_2_PO_4_ by sonication. HA-triGCN4 recombinant proteins from transiently transfected and stable Expi293F cells were purified by washing a column packed with Ni-NTA resin, which was incubated with HA-triGCN4 for 2 h at 4 °C, with 50 mM imidazole buffer and eluting with 300 mM imidazole buffer.

The plasmid encoding 4M2e-tetraGCN4 (GenScript) with a 6× His tag was generated by cloning into the pET21b vector with codon optimization. The 4M2e-tetraGCN4 recombinant protein was expressed by transforming into *E. coli* BL21 Star (DE3) (cat. #: C601003, Thermo Fisher Scientific). Expression was induced by adding isopropyl-β-d-1-thiogalactopyranoside (IPTG) at a final concentration of 1 mM when the optical density (OD600) reached 0.4–0.6. After incubation at 37 °C for 5 h at 200 rpm, the cells were harvested and lysed by 8 M urea buffer with 10 mM Tris-Cl and 100 mM NaH_2_PO_4_ at pH 8.0. The cell lysate was incubated with Ni-NTA for 2 h at 4 °C and washed with 8 M urea buffer at pH 6.3. 4M2e-tetraGCN4 was then collected by eluting with 8 M urea buffer at pH 5.9 and 4.5.

Following purification, HA-triGCN4 and 4M2e tetraGCN4 were buffer-exchanged into PBS (Corning) using a 3 kDa molecular weight cutoff Amicon Ultra-4 Centrifugal Filter Unit (Millipore Sigma). Endotoxin was removed from recombinant proteins using Pierce High Capacity Endotoxin Removal Spin Columns (Thermo Fisher Scientific) as per manufacturer’s instructions. Recombinant proteins were analyzed by SDS-PAGE and Western blot analysis. In brief, proteins were incubated with Laemmeli buffer solution (Biorad) containing 0.1 M dithiothreitol (DTT) at 95 °Cfor 5 min. The samples were then run through a 12% SDS-PAGE gel for 75 min at 150 V. The gel was stained with Coomassie Blue R-250, and for Western blot analysis, another gel was transferred to the membrane by applying 400 mA for 43 min at 4 °C. The membrane was blocked with 5% w/v dry milk and 0.1% Tween-20 in PBS, incubated with Penta-His Alexa Fluor 488 Conjugate (Qiagen, cat. #: 35310) antibody overnight, and then washed with 0.1% Tween-20 in PBS before imaging.

### LBL HA-4M2e Protein Nanoparticle Fabrication

For the synthesis of 4M2e NP cores, the desolvation method was utilized; 473 μL of 3.8 mg/mL 4M2e-tetraGCN4 was desolvated by adding 1.892 mL of 100% ethanol dropwise under constant mixing at 600 rpm. The desolvated 4M2e NPs were centrifuged at 21,000*g* for 15 min at room temperature and resuspended by sonication in 400 μL of PBS. 4M2e NPs were then stabilized by 18.7 μL of 10 μg/μL DTSSP while being stirred at 600 rpm for 1 h at 4 °C. Then, 4M2e NPs were biotinylated by incubating with 62 μL of 20 mM NHS-SS-PEG_4_-Biotin (Thermo Fisher Scientific) for 1 h at room temperature. To remove excess biotin, NPs were centrifuged at 21,000*g* for 25 min at 4 °C. After the supernatant was removed, the pellet was resuspended in 400 μL of PBS by sonication. For conjugation of HA to the biotinylated 4M2e NPs, the Avi-tag of HA was activated by using the BirA500 biotin protein ligase standard reaction kit (Avidity LLC) as per the manufacturer’s instructions. The activated biotinylated HA was then allowed to interact with SA10 streptavidin (Agilent) at a 1:1 molar ratio of HA/streptavidin for 30 min at room temperature. Afterward, 100 μg of the biotinylated 4M2e NPs was incubated with 400 μg of the mixture of HA and streptavidin for 1 h at room temperature. HA-4M2e NPs were centrifuged at 21,000*g* for 30 min at 4 °C, and the pelleted HA-4M2e NPs were resuspended in 200 μL of PBS by sonication. For LBL surface modification of HA-4M2e NPs, the same procedure was used as that for LBL OVA NPs. After the sequential coating process, LBL HA-4M2e NPs were subjected to a buffer exchange with PBS through centrifugation for excess polyelectrolyte removal.

### Hemagglutination Assay

The functional structure of HA was assessed by performing a hemagglutination assay. Soluble HA, HA-4M2e NPs, and LBL HA-4M2e NPs at a concentration of 500 μg/mL were serially diluted by twofold. Then, 50 μL of each protein or NP in a round-bottom 96-well plate was mixed with 50 μL of 0.75% turkey red blood cells (RBCs) (cat. #: 7249409, Lampire Biological Laboratories). The mixture was incubated for 1 h at room temperature to develop. Any well that did not display a red dot of settled RBCs was identified to induce hemagglutination.

### Animal Handling

Six to eight week old BALB/c mice (Charles Rivers) were maintained under pathogen-free conditions in individually ventilated and watered cages kept at negative pressure. Mice were kept in rooms on a 12 h light/dark cycle with ambient temperature between 22.8 and 23.9 °C with 30–40% relative humidity. Food was provided to mice *ad libitum* with only alfalfa-free food for the biodistribution animals. Animals were acclimatized for at least 6 days before the beginning of the experiments. Animals were randomly distributed among the experimental groups. All materials were tested for endotoxin levels using a ToxinSensor Chromogenic LAL Endotoxin Assay Kit (GenScript) to ensure an endotoxin limit under 15 EU/mg.

At the end of each animal study, animals were sacrificed by CO_2_ asphyxiation for biodistribution or a ketamine/xylazine/acepromazine cocktail for immune response. All animals were cared for according to the Georgia Institute of Technology Physiological Research Laboratory policies and under ethical guidance from the university’s Institutional Animal Care and Use Committee following National Institutes of Health (NIH) guidelines associated with the protocol number A100235. Vaccinated animals were transported to Georgia State University for challenge studies, performed under ethical guidance from the university’s Institutional Animal Care and Use Committee associated with the protocol number A23049.

### Distribution in Mice Nasal Cavity

Six to eight week old BALB/c mice (10 male and 10 female per formulation) were loaded into a customized physical restraint cylinder with an open nose cone end. Intranasal administration was conducted by slowly pipetting 5 μg of fluorescently labeled OVA, PEG OVA NPs, and LBL OVA NPs in 25 μL of saline (0.9% sodium chloride) dropwise in the bilateral nares. Mice (*n* = 5) were anesthetized with 3–5% isoflurane under a black paper before imaging. Mice were imaged by the IVIS Spectrum in vivo fluorescence imaging system (PerkinElmer) to confirm the distribution of NPs in the nasal cavity at time intervals between 30 min and 48 h after intranasal administration.

### NALT and Lung Histology

Mice were sacrificed at different time points for each group (*n* = 5) after IN administration. For tissue preparation, the head and skin were removed using 4-1/2″L scissors. The top skull and nasal cavity were placed in a solution of 3.7% formaldehyde (Fisher Scientific, histological grade) for 12 h at 4 °C covered from light. The tissue was washed and placed in a decalcification neutral formalin buffer with 1:1 solution of 8% hydrochloric acid (Sigma-Aldrich) and 8% formic acid stock solution (Sigma-Aldrich) 20 times the tissue volume for 24 h. Specimens were transferred to a 0.3% ammonia hydroxide solution to neutralize acids left in specimens for 30 min and washed extensively with deionized water. Finally, specimens were placed in 30% sucrose in cell culture grade PBS (Corning) overnight, covered from light. Lungs were collected and placed into a solution of 3.7% formaldehyde overnight at 4 °C, covered from light, and transferred into a solution of 30% sucrose. Samples were placed into Peel-A-Way embedding molds (Thermo Fisher Scientific) with the Scigen Tissue-Plus O.C.T. Compound (Thermo Fisher Scientific). Embedded tissue was then placed under a vacuum to remove bubbles and flash frozen with liquid nitrogen while being insulated with 2-methylbutane (TCI Chemicals). Frozen tissue blocks were stored at −70 °C. Sections were produced using a CryoStar NX50 Cryostat (Thermo Fisher Scientific) fitted with Gold Microtome Blades (C.L. Sturkey). The initial tissue point was measured, and sections were cut to a 14 μm thickness. Each section was collected on Superfrost Plus Gold Slides (Electron Microscopy Sciences) and stored at −20 °C prior to immunofluorescence staining.

For immunofluorescence staining, sections were washed gently with PBS to remove the O.C.T. Compound three times. Tissue was outlined using an ImmEDGE Hydrophobic Barrier Pen (Vector Laboratories) and blocked using a blocking buffer (2% BSA in PBS with 0.1% Tween-20) for 1 h at room temperature in a humidity chamber. Tissues were stained using the 1:150 antimouse CD19 polyclonal antibody (Invitrogen, cat. #: PA5-114969) in the blocking buffer overnight at 4 °C in a humidity chamber and washed three times with PBS. Samples were then blocked with 10% goat serum (Invitrogen) for 1 h at room temperature and washed three times with PBS. To stain samples, the Tyramide SuperBoost Kit Alexa Fluor 555 (Invitrogen) was used. Briefly, samples were incubated with the poly-HRP-conjugated secondary antibody overnight at 4 °C in a humidity chamber. Samples were washed three times with PBS at room temperature. The tyramide working solution (100 μL) was added for 6 min and quenched with the stop reagent solution. Blocking was performed using 2% BSA in PBS with 0.1% Tween-20 for 1 h at room temperature in a humidity chamber. The 1:150 antimouse CD11b Polyclonal antibody (Invitrogen, cat. #: PIPA579532) in the blocking buffer was added overnight at 4 °C in a humidity chamber and washed three times with PBS. Secondary antibody labeling was achieved using the Tyramide SuperBoost Kit Alexa Fluor 488 (Invitrogen). The slides were washed four times with PBS (5 min each), incubated with DAPI for 5 min at room temperature, and mounted using the ProLong Gold Antifade Reagent (Life Technologies). High-resolution multispectral imaging of the sections was conducted using a Vectra Polaris multispectral imaging system (Akoya Biosciences).

### Flow Cytometry NALT Uptake

After 6 h from IN administration, four mice per group were sacrificed by CO_2_ asphyxiation. NALT was isolated by the removal of the lower jaw, and a no. 11 surgical blade was used to excise the upper palate using the inside contour of the mouse incisor as reference. NALT was then isolated and placed into prewarmed Roswell Park Memorial Institute (RPMI) 1640 culture medium (37 °C) supplemented with HEPES (Gibco), l-glutamine (Gibco), and 10% FBS (Gibco). NALT was resuspended into a single cell culture using mechanical dissociation with 70 μm strainers (Cole-Parmer) with a 1 mL syringe plunger to obtain single cells. NALT on the strainer was rinsed with 5 mL of complete RPMI culture medium, and single cells were collected by centrifugation at 350*g* and 4 °C for 5 min. A total of 1 × 10^6^ single cells were seeded in each well of a round-bottom 96-well plate (Thermo Fisher Scientific), centrifuged at 350*g* and 4 °C for 5 min, and resuspended in 100 μL of complete RPMI. The Trustain FcX Plus (antimouse CD16/32) blocking buffer (BioLegend, cat. #: 156604) was added to block Fc receptors on the cell surface and washed with 1% bovine serum albumin (BSA, Thermo Fisher Scientific) in PBS. Cells were stained using 2 μL of FITC antimouse CD11b (Biolegend, cat. #: 101206) and 2 μL of PE antimouse CD19 (BioLegend, cat. #: 152408). Stained cells were washed again with 1% BSA and fixed with 3.7% formaldehyde (Thermo Fisher Scientific) for 30–40 min. Finally, cells were resuspended in 1% BSA PBS and analyzed by CytoFLEX S (Beckman Coulter).

### Animal Immunization Study

Six BALB/C mice (three male and three female, 6 to 8 weeks old) per group were administered IN with 5 μg of soluble OVA, OVA NPs, or PEG OVA NPs or 13 μg of LBL OVA NPs (5 μg of OVA, 3.5 μg of CpG ODN 1826, and 4.5 μg of trimethyl chitosan) using a physical restraint cylinder with an open nose cone end and boosted 4 weeks later. For the immunization study with influenza vaccine NPs, five BALB/C mice (five female, 6 to 8 weeks old) per group were administered with two doses of soluble mixture of 2.5 μg HA and 2.5 μg 4M2e with 3.5 μg CpG ODN 1826 and 4.5 μg trimethyl chitosan, 5 μg of HA-4M2e NPs, or 13 μg LBL HA-4M2e NPs (2.5 μg HA, 2.5 μg 4M2e, 3.5 μg CpG ODN 1826, and 4.5 μg trimethyl chitosan) IM, IN, or IM prime followed by IN boost.

Blood samples were withdrawn from the jugular vein of 3–5% isoflurane anesthetized mice at weeks 3 and 7. For the assessment of memory response, blood was collected at weeks 12, 16, and 20. Collected blood samples were allowed to clot at room temperature for 30 min and centrifuged in microtainer capillary blood collectors (BD) at 6000*g* for 5 min to isolate sera. On the 8th or 20th week, mice were euthanized by a ketamine/xylazine cocktail via intraperitoneal injection followed by intramuscular injection of a second dose in thigh muscles of the hind limb. BAL fluid was collected using methods described in Van Hoecke et al.^117^ Spleens, lungs, and subiliac lymph nodes were collected in a prewarmed RPMI 1640 medium supplemented with 10% FBS.

### ELISA for Antibody Titer Measurement

ELISA was performed to determine the titers of the OVA-, HA-, and 4M2e-specific antibody in sera using the following method. Nunc Maxisorp 96-well immune assay plates were coated with 1 μg/mL of OVA, H1N1 HA (strain A/California/04/2009), and 4M2e in PBS at room temperature overnight. To determine PEG- and chitosan-specific antibody titers in sera, Nunc Maxisorp 96-well immune assay plates were coated with 1 μg/mL BSA conjugated to *O*-[(*N*-succinimidyl)succinyl-aminoethyl]-*O*′-methylpolyethylene glycol with average Mn 750 or chitosan in PBS at room temperature overnight. Each well was blocked with 200 μL of 1% BSA in PBS at room temperature for 2 h. After washing the OVA, HA, and 4M2e coated plates with PBS with 0.05% Tween 20, each well was incubated with serially diluted sera at room temperature for 1 h. To wash PEG and chitosan conjugated plates, PBS with 0.05% CHAPS (3-((3-cholamidopropyl)dimethylammonio)-1-propanesulfonate) (Sigma-Aldrich) was used as a washing buffer for anti-PEG antibody response and 1% BSA in PBS for antichitosan antibody. One hundred microliters of HRP-conjugated goat antimouse IgG (Southern Biotech, cat. #: 1036-05), IgG1 (Southern Biotech, cat. #: 1071-05), IgG2a (Southern Biotech, cat. #: 1081-05), or IgA (Southern Biotech, cat. #: 1040-05) with 1:5000 dilution was then added to each well. After incubation at room temperature for 1 h, each well was washed three times with PBS with 0.05% Tween 20 followed by the addition of 50 μL of the TMB Chromogen Solution (Thermo Fisher Scientific) to each well. The enzymatic activity of HRP was stopped by 50 μL of the ELISA Stop Solution (Invitrogen) in each well, and the optical density (OD) signals at 450 and 570 nm were measured. The end point titer of antibodies from each mouse was determined by calculating the reciprocal of the highest dilution that gives OD_450–570 nm_ twice that of the prevaccination sera at the same dilution. For the measurement of IgG and IgA titers secreted from bone marrow cells of vaccinated mice, 5 × 10^6^ bone marrow cells/mL in each well were stimulated with R848/IL-2 (B-Poly-S reagent) as described in the B-Cell ELISpot Assay, and 200 μL of the supernatant from each well was collected for ELISA after centrifuging the bone marrow cells in a round-bottom 96-well plate at 400*g* for 5 min.

### Intracellular Cytokine Staining

Harvested spleens and lymph nodes were strained through 70 μm strainers with a 1 mL syringe plunger to obtain single cells. The spleens or lymph nodes on the strainers were then rinsed with 10 mL of complete RPMI 1640 medium (supplemented with 8 mM HEPES (Gibco), 50 μM 2-mecaptoethanol (Gibco), and 2 mM l-glutamine (Gibco)) and 10% FBS, and single cells were centrifuged at 350*g* and 4 °C for 5 min. Lymph node cell pellets were resuspended in 200 μL of complete RPMI 1640 medium, while splenocytes were incubated in 1 mL of ACK lysing (Thermo Fisher Scientific) buffer for 9 min, followed by quenching the lysis with 9 mL of complete RPMI 1640 medium. Splenocytes were then centrifuged at 350*g* and 4 °C for 5 min and resuspended in 5 mL of complete RPMI 1640 medium. Lungs harvested from vaccinated mice were processed into cell suspensions with a gentle MACS Octo Dissociator and Lung Dissociation Kit (Miltenyi Biotec) according to the manufacturer’s instructions. Cells were run through 70 μm strainers to ensure the presence of single cells. Cells were centrifuged at 500*g* for 5 min, resuspended at 1 × 10^6^ cells/mL in complete RPMI 1640 medium, and plated in a round-bottom 96-well plate.

For staining intracellular cytokines, 1 × 10^6^ single cells were seeded in each well of a round-bottom 96-well plate, centrifuged at 350*g* and 4 °C for 5 min, and resuspended in 100 μL of complete RPMI 1640 medium. To restimulate OVA-, HA-, or M2e-specific T cells, 2 μL of reconstituted PepTivator Ovalbumin or PepTivator H1N1 HA (strain A/California/04/2009) (Miltenyi Biotec) solution or 10 μg/mL M2e human consensus peptide (Synpeptide, Seq: SLLTEVETPIRNEWGSRSN) was added to each well containing 1 × 10^6^ single cells. For positive controls, 0.5 μL of 2 μg/mL phorbol myristate acetate and 0.5 μL of 100 μg/mL calcium ionophore (TLC grade, ≥98%, Millipore Sigma) were added to each well containing 1 × 10^6^ single cells. After incubation at 37 °C for 3 h, 2 μL of a mixture of 50× brefeldin A (Biolegend) and 50× monensin (Biolegend) was added to each well to retain cytokines within the endoplasmic reticulum during cell activation for another 3 h.

Before staining cellular surface markers, T cells and memory B cells were stained with a Zombie Violet Viability Kit (Biolegend, cat. #: 423113) and a LIVE/DEAD Fixable Aqua Dead Stain Kit (Thermo Fisher Scientific, cat. #: L34965) for 35 min, respectively. Cells were resuspended in 0.5 μL/10^6^ cells in Trustain FcX Plus (Biolegend, cat. #: 156604) blocking buffer to block Fc receptors on the cell surface. Cells were then centrifuged and washed with 1% BSA in PBS. For staining T cells, cells were incubated with 2 μL/10^6^ cells PerCP anti-CD3 (Biolegend, cat. #: 100326), 1 μL/10^6^ cells FITC anti-CD8 (Biolegend, cat. #: 100706), and 1 μL/10^6^ cells APC/Cy7 anti-CD4 (Biolegend, cat. #: 100525) for 40 min. For the assessment of memory B cells, cells harvested from lymph nodes were stained with 2 μL/10^6^ PerCP anti-CD3 (Biolegend, cat. #: 100326), 1.25 μL/10^6^ cells PerCP/Cy5.5 anti-B220 (Biolegend, cat. #: 103235), 0.5 μL/10^6^ cells AlexaFluor 700 anti-IgD (Biolegend, cat. #: 405729), 1.25 μL/10^6^ cells APC anti-CD273/PD-L2 (Biolegend, cat. #: 107210), and 5 μL/10^6^ cells Brilliant Violet 605 anti-CD73 (Biolegend, cat. #: 127215). Stained cells were washed again with 1% BSA and fixed with 3.7% formaldehyde for 20 min at room temperature. To stain intracellular cytokines of restimulated T cells, cells were resuspended in 1× permeabilization buffer (eBioscience) with 1.5 μL/10^6^ cells PE anti-IFN-γ (Biolegend, cat. #: 505808) and 5 μL/10^6^ cells PE/Cy7 anti-IL-4 (Biolegend, cat. #: 504118). Finally, all T cells and memory B cells were resuspended in 1% BSA and analyzed by Cytek Aurora flow cytometry (Cytek Biosciences).

### B-Cell ELISpot Assay

Bone marrow cells were collected by flushing the femurs and tibiae with complete RPMI 1640 medium. The cells were then strained through 70 μm strainers to obtain single cells and resuspended in complete RPMI 1640 medium at 5 × 10^6^ cells/mL. A B-ELISpot assay was performed to analyze the number of anti-HA and anti-4M2e memory B cells from the bone marrow cells using Mouse IgA/IgG Double-Color ELISPOT (ImmunoSpot) as per the manufacturer’s instructions. Briefly, 5 × 10^6^ cells/mL of bone marrow cells were stimulated by incubating them with a mixture of the TLR7/8 agonist R848 at 1 μg/mL and recombinant human IL-2 at 10 ng/mL (B-Poly-S reagent, ImmunoSpot) for 72 h in complete RPMI media under humidified conditions at 37 °C and 5% CO_2_ atmosphere. A 96-well, high-protein-binding PVDF filter plate was prewetted using 70% ethanol to each well and rapidly washed with cell culture grade PBS. The plate was washed with PBS and coated with 1 μg/well of either HA-triGCN4 or 4M2e-tetraGCN4 overnight in the capture solution (ImmunoSpot) in a humid chamber at 4 °C. The antigen-coated PVDF plate was washed twice with 150 μL of PBS to remove excess unbound antigen. A total of 100 μL/well of 2 × 10^6^ cells were seeded on the plate and incubated at room temperature for 1 h followed by incubation at 37 °C and 5% CO_2_ overnight. Cells were then removed, and the plate was washed twice with PBS. The plate was incubated with 80 μL/well of antimouse IgA/IgG Detection Solution (ImmunoSpot) for 2 h in the dark at room temperature. The plate was washed twice with PBS supplemented with Tween-20 (0.05%) and incubated with 80 μL/well of tertiary solution (streptavidin, alkaline phosphatase, and FITC-horseradish peroxidase) (ImmunoSpot) for 1 h in the dark at room temperature. The plate was washed with deionized water followed by color development of blue using the CTL-TrueBlue Substrate (ImmunoSpot) for 10 min. After the plate was washed with deionized water, red color development was achieved using the CTL-TrueRed Substrate Solution (ImmunoSpot) for 7 min in the dark. The plate was then washed with deionized water and dried in the dark overnight. The developed spots were imaged and analyzed by a BioTek Cytation 7 (Agilent). The percent population of antigen-specific IgG (blue spot) and IgA (red spot) secreting B cells was reported by calculating the spot forming unit (SFU) of antigen-specific B cells per SFU of the total B cells.

### T-Cell ELISpot Assay

A T-cell ELISpot assay was performed to assess HA- and 4M2e-specific T-cell responses at week 20 by using Mouse IFN-γ/IL-2 Double-Color ELISPOT (ImmunoSpot) as per the manufacturer’s instructions. Briefly, 4 × 10^5^ cells/well of isolated splenocytes were incubated with HA-triGCN4 or 4M2e-tetraGCN4 at a final concentration of 10 μg/mL in the CTL-Test Medium for 24 h at 37 °C and 5% CO_2_ atmosphere. On the next day, IFN-γ and IL-2 spots were detected by 80 μL/well of antimouse IFN-γ/IL-2 detection solution and revealed after incubation with 80 μL/well of tertiary solution, 80 μL/well of blue developer solution, and 80 μL/well of red developer solution. The reaction was then stopped by washing the membrane with tap water. After the plate was dried in the dark overnight, the blue and red spots were imaged and analyzed by a BioTek Cytation 7 (Agilent).

### Virus Challenge and Lung Viral Titration

At 6 weeks after boost vaccination, the first set vaccine and control groups (*n* = 5 BALB/c mice) via IN/IN and IM/IM delivery were intranasally challenged with A/California/04/2009 H1N1 virus (4× LD_50_) and monitored daily to measure body weight changes and survival rates. At 75 days after boost dose, the second set groups (*n* = 5) with the corresponding vaccine and routes were challenged with A/California/04/2009 H1N1 virus (3× LD_50_) and then sacrificed 8 days later to collect lung tissue samples. Lung extracts were prepared in 1.5 mL of RPMI 1640 media by mechanical grinding of lung tissues harvested and used to determine viral titers in Madin–Darby canine kidney (MDCK) cells. Briefly, MDCK cells were seeded in 96-well plates at a concentration of 4 × 10^4^ cells per well, and 10-fold serial dilutions of lung lysates from the infected mice were added to the cells. The plates were then incubated at 37 °C for 3 days. Following the 3 day incubation, a hemagglutination assay (HA) was performed by mixing equal volumes of harvested supernatant from the plates and 0.5% chicken red blood cells (RBCs, Lampire Biological Laboratories). Virus titers as 50% tissue culture infection dose (TCID_50_)/mL were evaluated according to the Reed and Muench method.^118^

### Statistical Analysis

For comparisons between more than two groups, one-way ANOVA was performed followed by Tukey’s *post hoc* multiple comparison analysis. Comparisons between groups with different routes of vaccination were analyzed by two-way ANOVA with Tukey’s *post hoc* multiple comparison analysis. Statistical significance was determined as follows: (*) for *p* ≤ 0.05, (**) for *p* ≤ 0.01, (***) for *p* ≤ 0.001, and (****) for *p* ≤ 0.0001. The statistical comparisons of survival rates between groups were analyzed by Kaplan–Meier with log rank test: ns for not significant, * for ≤0.033, and ** for ≤0.002 compared to the PBS control group. All data plotted with error bars are expressed as mean values with the standard deviation. The statistical analysis was performed with GraphPad Prism 9.

## Supporting Information Available

DLS data of OVA NP, PEG OVA NP, and LBL OVA NP and HEK-Blue hTLR9 assay; humoral immune responses induced by OVA + Adj, OVA NP, PEG OVA NP, and LBL OVA NP; SDS-PAGE and Western blot analysis of HA-triGCN4 and 4M2e-tetraGCN4; development of stable HEK293F cell lines expressing HA-triGCN4; humoral immune responses induced by HA + 4M2e + Adj, HA-4M2e NP, and LBL HA-4M2e NP after priming; gating strategy to identify activated T-cell subsets; cellular immune responses induced by HA + 4M2e + Adj, HA-4M2e NP, and LBL HA-4M2e NP; memory B-cell responses induced by HA + 4M2e + Adj, HA-4M2e NP, and LBL HA-4M2e NP; B-ELISpot images of HA- and 4M2e-specific IgG and IgA induced by HA + 4M2e + Adj, HA-4M2e NP, and LBL HA-4M2e NP; IgA and IgG titers from bone marrow cells at 16 weeks after boost; and amino acid sequence for recombinant proteins (PDF)
