# Supplementary material for: Multilayer Adjuvanted Influenza Protein Nanoparticles Improve Intranasal Delivery and Antigen-Specific Immunity
Source: ACS Nano. 2025 Feb 15;19(7):7005–25. doi: 10.1021/acsnano.4c14735 (PMC11867023; doi:10.1021/acsnano.4c14735)
Supplement: Supplementary file 1 — nn4c14735_si_001.pdf [file nn4c14735_si_001.pdf]

Supporting Information

Multilayer Adjuvanted Influenza Protein Nanoparticles Improve Intranasal Delivery and Antigen-Specific Immunity

Jaeyoung Park[‡ a], Thomas Pho[‡ a, b], Noopur Bhatnagar[c], Linh D. Mai [a], Mariela R. Rodriguez-Otero [a, b], Surya Sekhar Pal [c], Chau Thuy Tien Le [c], Sarah E. Jenison [a], Chenyu Li [a], Grace A. May [a], Marisa Arioka[d], Sang-Moo Kang[c], Julie A. Champion[a, b*]*

[a]School of Chemical and Biomolecular Engineering, Georgia Institute of Technology, Atlanta, GA, 30332, USA.

[b]Petit Institute for Bioengineering and Bioscience, Georgia Institute of Technology, Atlanta, GA, 30332, USA.

[c]Center for Inflammation, Immunity & Infection, Institute for Biomedical Sciences, Georgia State University, Atlanta, GA, 30302, USA

[d]Department of Chemistry, Tokyo University of Science, Tokyo, 162-8601, Japan

[*]co-corresponding author: julie.champion@chbe.gatech.edu (J.A.C), skang24@gsu.edu (S.K)

[‡]These authors contributed equally.

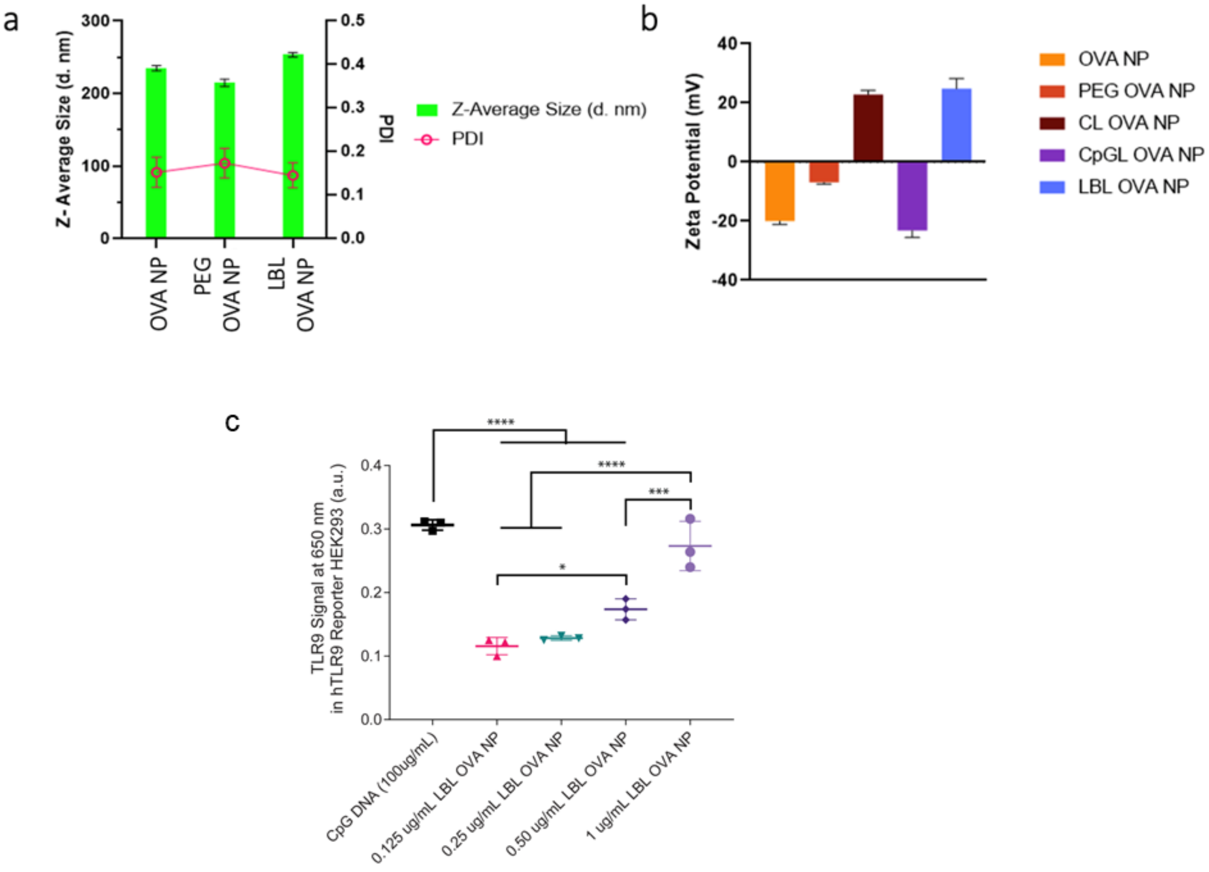

Figure S1. Characterization of OVA NPs. a,b) Hydrodynamic size measured by Dynamic Light Scattering (a) and zeta potential (b) measured by electrophoretic light scattering. c) HEK-Blue™ hTLR9 assay incubated with CpG ODN and LBL OVA NPs measured at 620 nm. 0.125 µg/mL LBL OVA NP contains 0.034 µg/mL CpG and 1 µg/mL contains 0.269 µg/mL CpG. P-values (n=3) were determined by one-way Anova with Turkey's post-hoc multiple comparison analysis: * for ≤ 0.05, *** for ≤ 0.001, **** for p ≤ 0.0001.

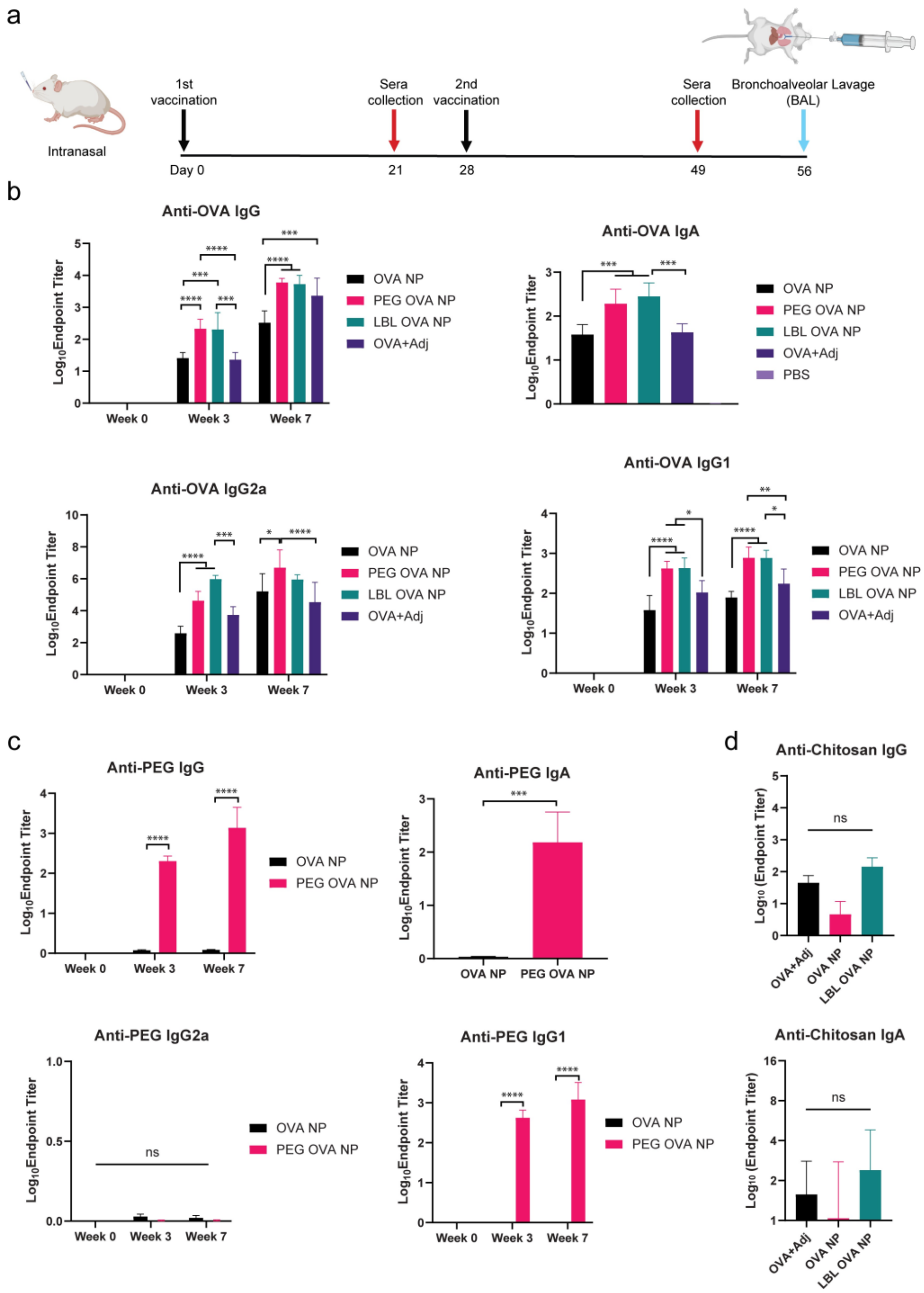

Figure S2. a) Timeline for vaccination and collection of sera and BAL fluid. b) Anti-OVA IgG, IgG2a, and IgG1 titers in sera and IgA titers in BAL fluid from mice intranasally immunized with OVA NP, PEG OVA NP, LBL OVA NP, and OVA+Adj. c) Anti-PEG IgG, IgA, IgG2a, and IgG1 titers from mice intranasally immunized with OVA NP and PEG OVA NP. d) Anti-chitosan IgG and IgA titers from mice intranasally immunized with OVA+Adj, OVA NP, and LBL-OVA NP. PBS-treated mice titers were below the limit of detection and are not shown. P-values (n=6) were

determined by one-way and two-way Anova with Turkey's post-hoc multiple comparison analysis: ns for not significant, * for ≤ 0.05, ** for ≤ 0.01, *** for ≤ 0.001, **** for p ≤ 0.0001.

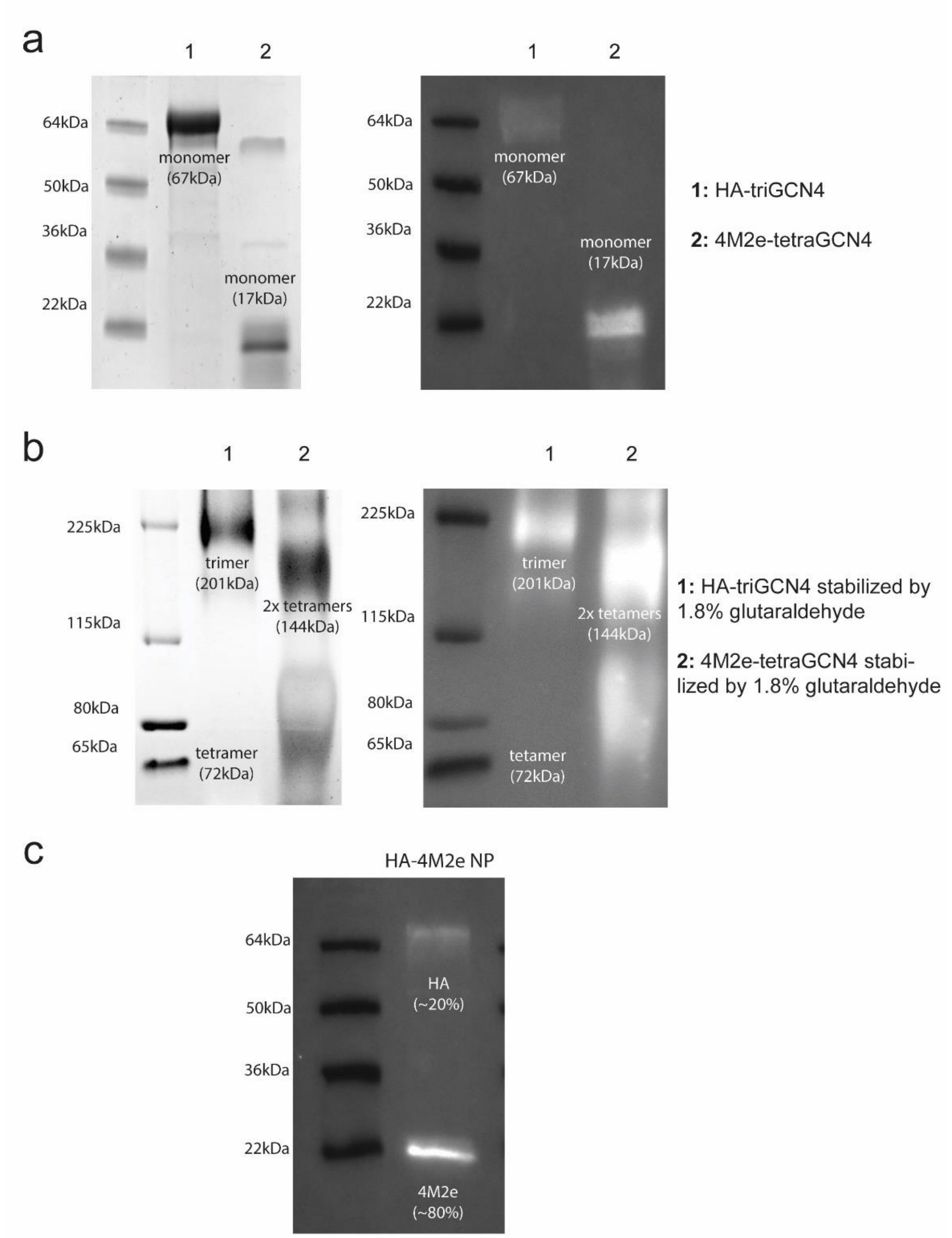

Figure S3. Analysis of antigens used for NP synthesis. a,b) SDS-PAGE and anti-Histag Western blot analysis of expressed HA-triGCN4 and 4M2e-tetraGCN4 monomers and trimeric HA-triGCN4 and tetrameric 4M2e-tetraGCN4 stabilized by 1.8% glutaraldehyde. c) Compositions of reduced HA-4M2e NPs analyzed by anti-Histag Western blot.

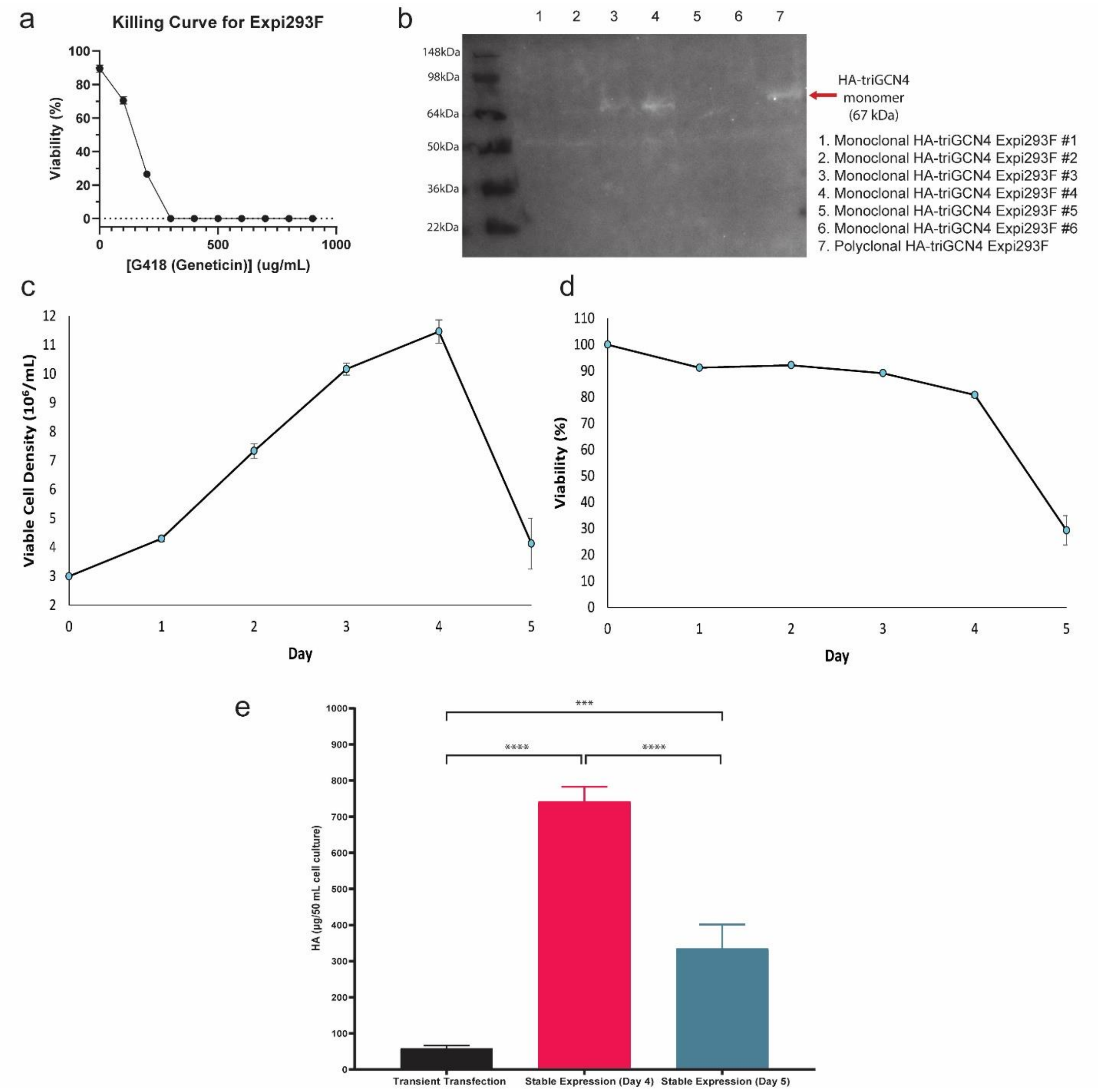

Figure S4. Development of stable HEK293F cell lines expressing HA-triGCN4. a) A killing curve to determine the optimum concentration of Geneticin at which non-transfected Expi293F cells are dead. b) Western blot analysis of HA-triGCN4 collected from lysed stable monoclonal Expi293F cells. c) A cell growth curve for stable Expi293F cells. d) Viability of stable Expi293F cells. e) Expression levels of HA-triGCN4 from transiently transfected and stable Expi293F cells. The amount of expressed HA-triGCN4 collected on day 4 was significantly higher than that on day 5. P-values (n=3) were determined by one-way Anova with Turkey's post-hoc multiple comparison analysis: * for ≤ 0.05, ** for ≤ 0.01, *** for ≤ 0.001, **** for p ≤ 0.0001.

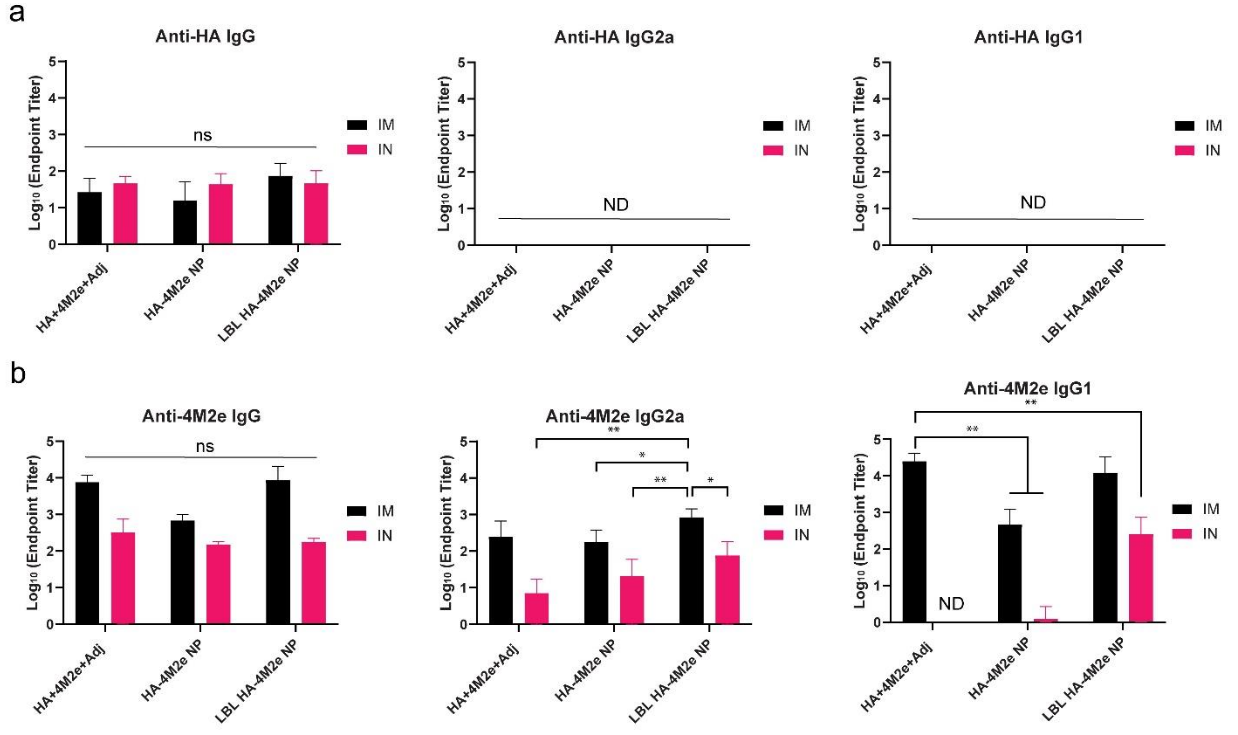

Figure S5. a,b) Anti-HA (a) and anti-4M2e (b) IgG, IgG2a, and IgG1 titers in sera collected on 21 days post-prime, prior to boost immunization, from mice immunized with HA+4M2e+Adj, HA-4M2e NP, and LBL HA-4M2e NP. PBS-treated mice titers were below the limit of detection and are not shown. ND stands for not detectable values. P-values (n=5) were determined by two-way Anova with Turkey's post-hoc multiple comparison analysis: * for ≤ 0.05, ** for ≤ 0.01.

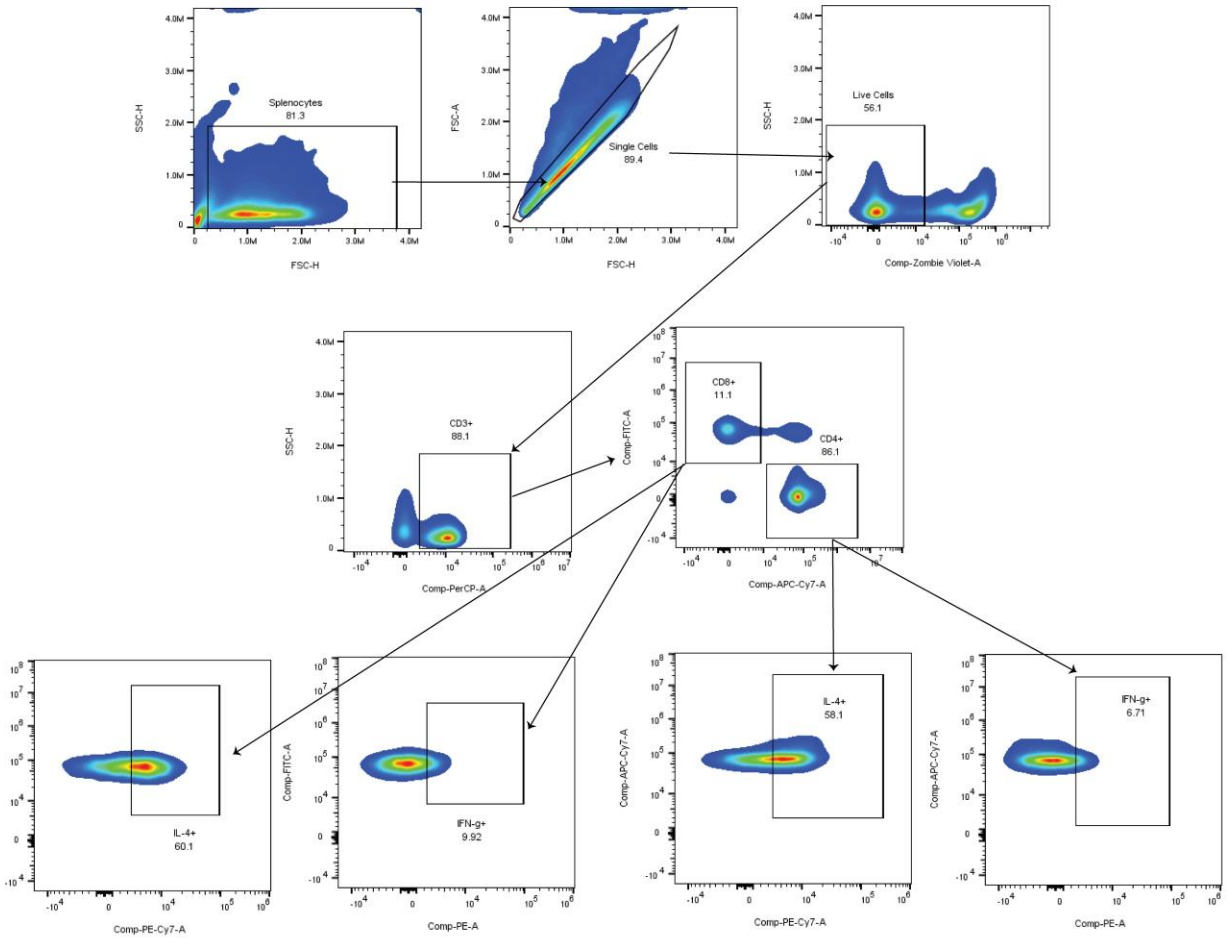

Figure S6. Gating strategy to identify activated T cell subsets.

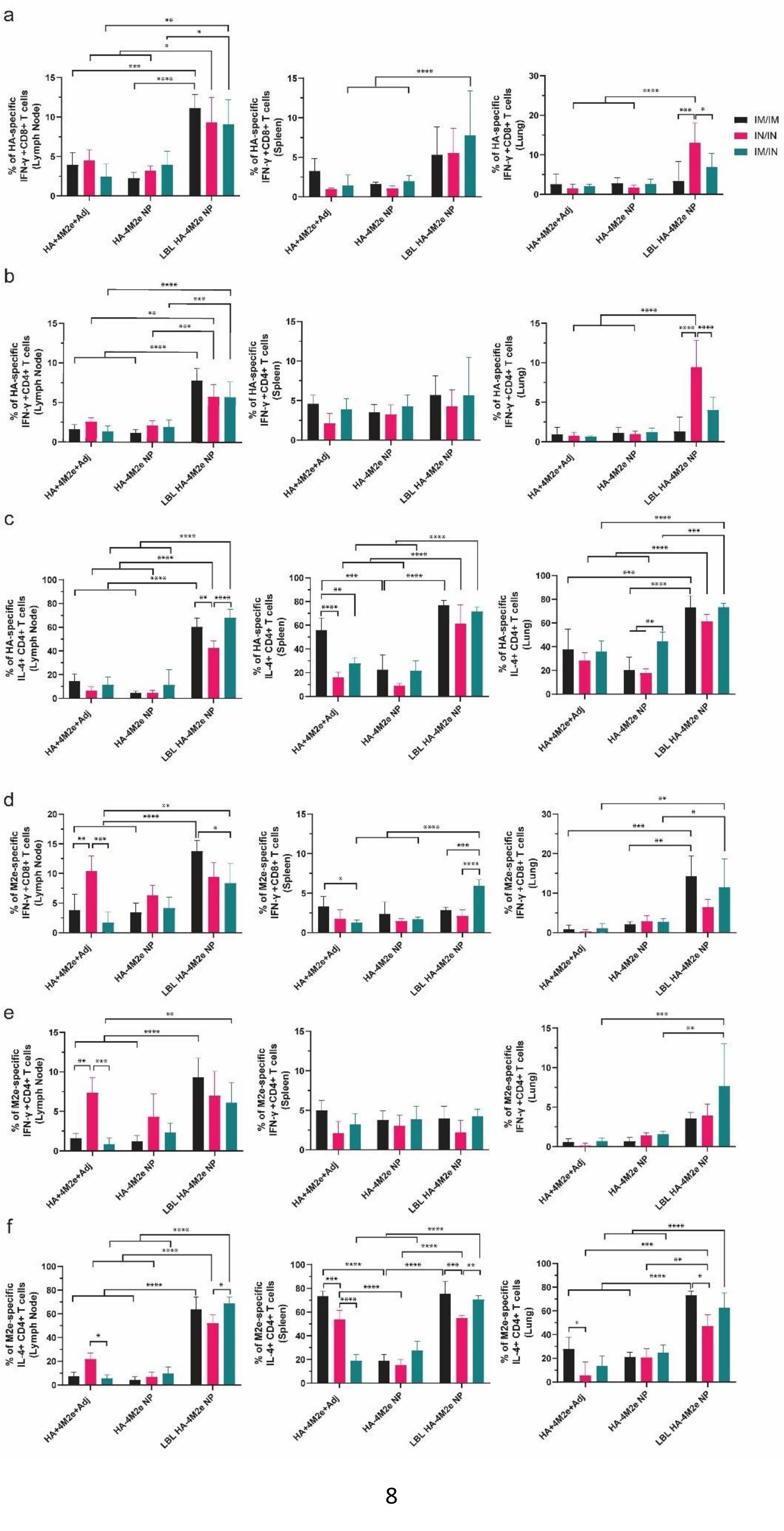
a
b
c
d
e
f
IM/IM
IN/IN
IM/IN
% of HA-specific IFN-γ +CD8+ T cells (Lymph Node)
% of HA-specific IFN-γ +CD8+ T cells (Spleen)
% of HA-specific IFN-γ +CD8+ T cells (Lung)
% of HA-specific IFN-γ +CD4+ T cells (Lymph Node)
% of HA-specific IFN-γ +CD4+ T cells (Spleen)
% of HA-specific IFN-γ +CD4+ T cells (Lung)
% of HA-specific IL-4+ CD4+ T cells (Lymph Node)
% of HA-specific IL-4+ CD4+ T cells (Spleen)
% of HA-specific IL-4+ CD4+ T cells (Lung)
% of M2e-specific IFN-γ +CD8+ T cells (Lymph Node)
% of M2e-specific IFN-γ +CD8+ T cells (Spleen)
% of M2e-specific IFN-γ +CD8+ T cells (Lung)
% of M2e-specific IFN-γ +CD4+ T cells (Lymph Node)
% of M2e-specific IFN-γ +CD4+ T cells (Spleen)
% of M2e-specific IFN-γ +CD4+ T cells (Lung)
% of M2e-specific IL-4+ CD4+ T cells (Lymph Node)
% of M2e-specific IL-4+ CD4+ T cells (Spleen)
% of M2e-specific IL-4+ CD4+ T cells (Lung)
HA+4M2e+Adj
HA-4M2e NP
LBL HA-4M2e NP

Figure S7. Antigen-specific cellular immune responses. a-c) Percent populations of restimulated HA-specific IFN-γ^+ $CD8^+$ T cells (a), IFN-γ^+ $CD4^+$ T cells (b), and IL-4^+ $CD4^+$ T cells (c) in lymph node, spleen, and lung. d-f) Percent populations of restimulated M2e-specific IFN-γ^+ $CD8^+$ T cells (d), IFN-γ^+ $CD4^+$ T cells (e), and IL-4^+ $CD4^+$ T cells (f) in lymph node, spleen, and lung. The population of activated T cells were analyzed by intracellular cytokine staining (ICS). Comparisons across groups are only shown for the same vaccination routes. P-values (n=5) were determined by two-way Anova with Tukey's post-hoc multiple comparison analysis: * for ≤ 0.05, ** for ≤ 0.01, *** for ≤ 0.001, **** for $p \leq 0.0001$.

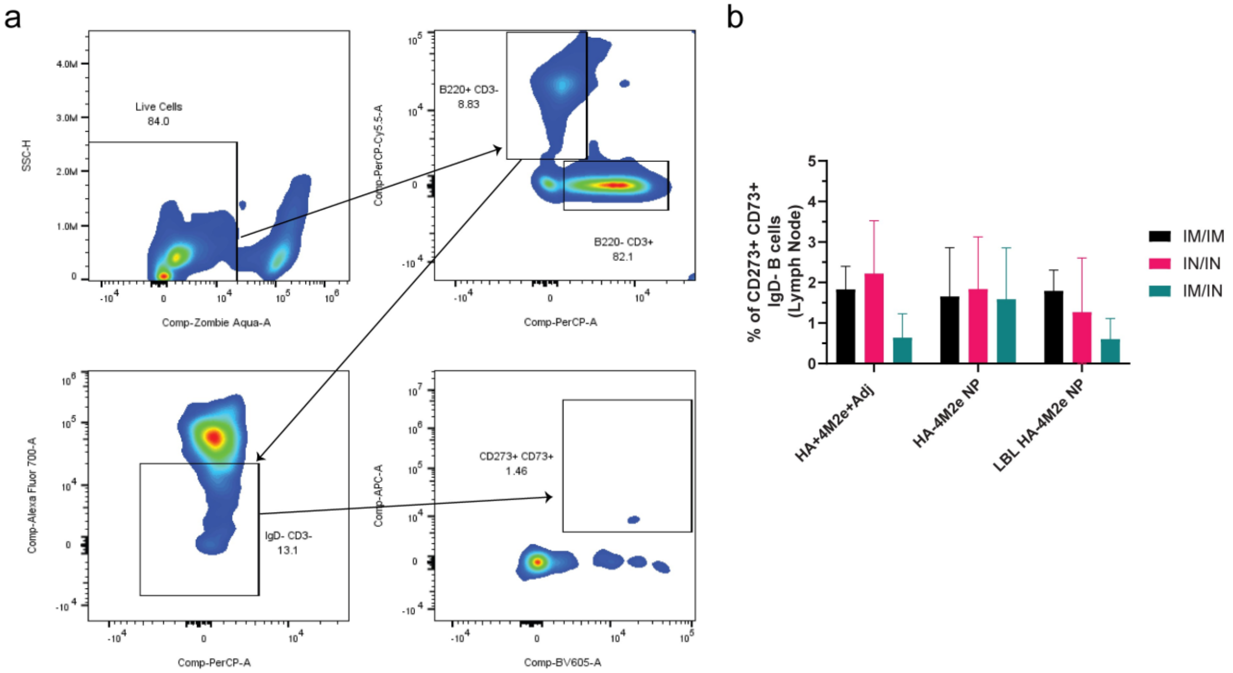

Figure S8. Memory B cell response in lymph node. a) Gating strategy to identify memory B cells. b) Total percent population of $CD273^+$ $CD73^+$ IgD^- memory B cells.

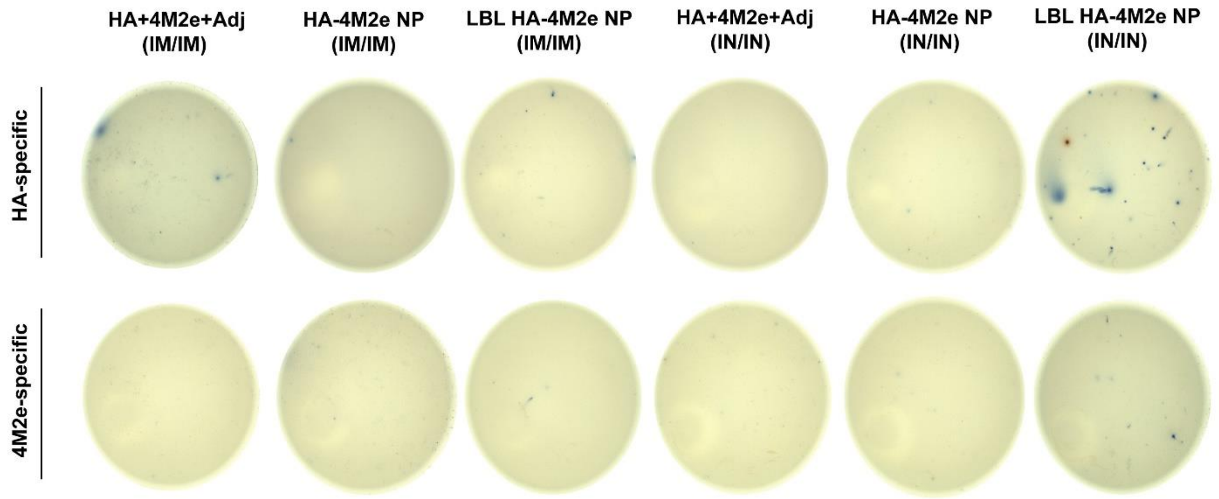

Figure S9. B-ELISpot images of HA- and 4M2e-specific IgG (blue spots) and IgA (red spots) secreting long-lived bone marrow B cells after stimulating with R848/IL-2 (B-Poly-STM reagent).

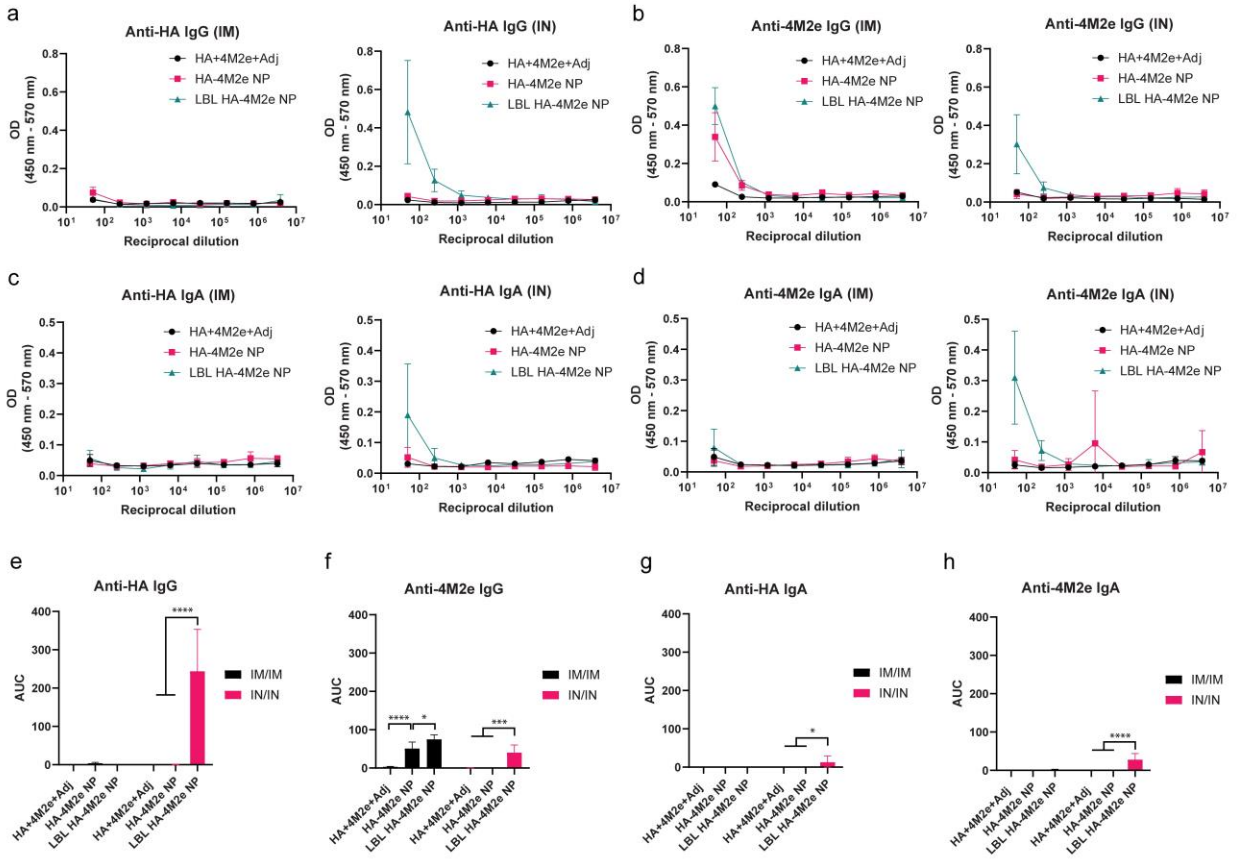

Figure S10. IgA and IgG titers from bone marrow cells stimulated with R848/IL-2 (B-Poly-S^{TM} reagent) at 16 weeks after boost. a-d) ELISA optical density (OD) difference for anti-HA IgG (a), anti-4M2e IgG (b), anti-HA IgA (c), and anti-4M2e IgA (d) secreted from bone marrow cells of mice vaccinated IM/IM and IN/IN with HA+4M2e+Adj, HA-4M2e NP, and LBL HA-4M2e NP. e-h) Area under the curve (AUC) for anti-HA IgG (e), anti-4M2e IgG (f), anti-HA IgA (g), and anti-4M2e IgA (h) from the same experiment as in (a-d). PBS-treated mice titers were below the limit of detection and are not shown. P-values (n=5) were determined by two-way Anova with Turkey's post-hoc multiple comparison analysis: * for ≤ 0.05, *** for ≤ 0.001, **** for p ≤ 0.0001.

**HA-triGCN4:** H1N1 HA-trimeric GCN4-Avitag-His tag (67 kDa)	MKAILVVLLYTFATANADTLCIGYHANNSTDTVDTVLEKNVTVTHSVNLLEDKHNGKLCKLRGVAPLHLGKCNIAGWILGNPECESLSTASSWSYIVETPSSDNGTCYPGDFIDYEELREQLSSVSSFERFEIFPKTSSWPNHDSNKGVTAACPHAGAKSFYKNLIWLVKKGNSYPKLSKSYINDKGKEVLVLWGIHHPSTSADQQSLYQNADTYVFVGSSRYSKKFKPEIAIRPKVRDQEGRMNYYWTLVEPGDKITFEATGNLVVPRYAFAMERNAGSGIIISDTPVHDCNTTCQTPKGAINTSLPFQNIHPITIGKCPKYVKSTKLRLATGLRNIPSIQSRGLFGAIAGFIEGGWTGMVDGWYGYHHQNEQGSGYAADLKSTQNAIDEITNKVNSVIEKMNTQFTAVGKEFNHLEKRIENLNKKVDDGFLDIWTYNAELLVLLENERTLDYHDSNVKNLYEKVRSQLKNNAKEIGNGCFEFYHKCDNTCMESVKNGTYDYPKYSEEAKLNREEIDGVPGSRMKQIEDKIEEILSKIYHIENEIARIKKLVGERGSGSENLYFQGSAGGLNDIFEAQKIEWHEGSGSHHHHHH
**4M2e-tetraGCN4:** Human M2e-Swine M2e-Avian M2e-Fowl M2e-tetrameric GCN4-Histag (17 kDa)	SLLTEVETPIRNEWGSRSNDSSDPGGSSGGSSSLLTEVETPTRSEWESRSSDSSDPGGSSGGSSSLLTEVETPTRNGWESKSSGSSDPGSGSGSGSSLLTEVETPTRNGWESNSSDSSDPGGGGSSSSLELKQIEDKLEEILSKLYHIENELARIKKLLGEHHHHHH

Table S1. Amino acid sequence for recombinant proteins, HA-triGCN4 and 4M2e-tetraGCN4, used to form HA-4M2e NPs.
